# Oncogenic Kras^G12D^ causes myeloproliferation via NLRP3 inflammasome activation

**DOI:** 10.1038/s41467-020-15497-1

**Published:** 2020-04-03

**Authors:** Shaima’a Hamarsheh, Lena Osswald, Benedikt S. Saller, Susanne Unger, Donatella De Feo, Janaki Manoja Vinnakota, Martina Konantz, Franziska M. Uhl, Heiko Becker, Michael Lübbert, Khalid Shoumariyeh, Christoph Schürch, Geoffroy Andrieux, Nils Venhoff, Annette Schmitt-Graeff, Sandra Duquesne, Dietmar Pfeifer, Matthew A. Cooper, Claudia Lengerke, Melanie Boerries, Justus Duyster, Charlotte M. Niemeyer, Miriam Erlacher, Bruce R. Blazar, Burkard Becher, Olaf Groß, Tilman Brummer, Robert Zeiser

**Affiliations:** 1 Department of Medicine I, Medical Center - University of Freiburg, Faculty of Medicine, University of Freiburg, Freiburg, Germany; 2 Faculty of Biology, University of Freiburg, Freiburg, Germany; 3 Institute of Neuropathology, University Medical Center Freiburg, Faculty of Medicine, University of Freiburg, Freiburg, Germany; 4 Institute of Experimental Immunology, University of Zurich, Zurich, Switzerland; 5 Department of Biomedicine, University of Basel and University Hospital Basel, Basel, Switzerland; 6 Institute of Medical Bioinformatics and Systems Medicine, Medical Center - University of Freiburg, Faculty of Medicine, University of Freiburg, Freiburg, Germany; 7 Clinic for Rheumatology and Clinical Immunology, University Medical Center Freiburg, Faculty of Medicine, University of Freiburg, Freiburg, Germany; 8 University of Freiburg, Freiburg, Germany; 9 Institute for Molecular Bioscience, University of Queensland, Brisbane, Australia; 10 German Cancer Consortium (DKTK) Partner Site Freiburg, German Cancer Research Center (DKFZ), Heidelberg, Germany; 11 Comprehensive Cancer Centre Freiburg (CCCF), University of Freiburg, Freiburg, Germany; 12 Division of Pediatric Hematology and Oncology, Department of Pediatrics and Adolescent Medicine, University Medical Center Freiburg, Faculty of Medicine, University of Freiburg, Freiburg, Germany; 13 Division of Blood and Marrow Transplantation, Department of Pediatrics, Masonic Cancer Center, University of Minnesota, Minneapolis, MN 55455 USA; 14 Centre for Biological Signalling Studies (BIOSS) and Centre for Integrative Biological Signalling Studies (CIBSS), University of Freiburg, Freiburg, Germany; 15 Institute of Molecular Medicine and Cell Research (IMMZ), Faculty of Medicine, University of Freiburg, Freiburg, Germany; 16 Centre for Biological Signalling Studies (BIOSS), University of Freiburg, Freiburg, Germany

**Keywords:** Targeted therapies, Bone marrow transplantation, NOD-like receptors, Myeloproliferative disease

## Abstract

Oncogenic *Ras* mutations occur in various leukemias. It was unclear if, besides the direct transforming effect via constant RAS/MEK/ERK signaling, an inflammation-related effect of KRAS contributes to the disease. Here, we identify a functional link between oncogenic *Kras*^*G12D*^ and NLRP3 inflammasome activation in murine and human cells. Mice expressing active *Kras*^*G12D*^ in the hematopoietic system developed myeloproliferation and cytopenia, which is reversed in *Kras*^*G12D*^ mice lacking NLRP3 in the hematopoietic system. Therapeutic IL-1-receptor blockade or NLRP3-inhibition reduces myeloproliferation and improves hematopoiesis. Mechanistically, Kras^G12D^-RAC1 activation induces reactive oxygen species (ROS) production causing NLRP3 inflammasome-activation. In agreement with our observations in mice, patient-derived myeloid leukemia cells exhibit KRAS/RAC1/ROS/NLRP3/IL-1β axis activity. Our findings indicate that oncogenic KRAS not only act via its canonical oncogenic driver function, but also enhances the activation of the pro-inflammatory RAC1/ROS/NLRP3/IL-1β axis. This paves the way for a therapeutic approach based on immune modulation via NLRP3 blockade in KRAS-mutant myeloid malignancies.

## Abstract (web summary)

Oncogenic *Ras* mutations are common drivers in myeloid leukemia. Here, the authors show in patient cells and in mice that oncogenic K-Ras activates NLRP3 inflammasome to drive myeloproliferation, which can be reversed by genetic or pharmacologic NLRP3 blockade.

## Introduction

Activating mutations of *NRAS* and *KRAS* genes were reported to occur in 18–32% of acute myeloid leukemia (AML)^1,2^, in 11–38% of chronic myelomonocytic leukemia (CMML)^3,4^ and in 25–35% of juvenile myelomonocytic leukemia (JMML) patients^5,6^. JMML is an aggressive myeloproliferative disease (MPD) of early childhood characterized clinically by the overproduction of myelomonocytic cells^7^. Other mutations found in this disease include mutations in the tumor suppressor gene *NF1*^8^, CBL and mutations in *PTPN11*^9^, which also activate the RAS/extracellular-signal-regulated kinase (ERK) signaling pathway^10^, reviewed in ref. ^11^. CMML is a clonal stem cell disorder associated with peripheral blood monocytosis, myeloid cell proliferation with myeloid cell dysplasia and ineffective hematopoiesis, and can transform to AML^12^.

Since the RAS/RAF/MEK/ERK cascade couples signals from cell surface receptors to transcription factors in non-malignant immune cells^13^, we hypothesized that in addition to the oncogenic signal, inflammatory mechanisms may drive the proliferation of myeloid cells and cause fever, wasting syndrome and failure to thrive, which is frequently observed in JMML, CMML and more rarely in AML patients. This hypothesis was also based on our previous work showing that signaling caused by FLT3-ITD and JAK2-V617F oncoproteins can affect the expression of the inflammation-related molecules IL-15^14^ and PD-L1^15^, respectively. In support of the concept that RAS/ERK signaling plays a role in inflammation, recent studies had shown that ERK1/2 phosphorylation is critical for allogeneic immune activation during graft-versus-host disease (GVHD)^16^ and MEK inhibition blocked these pro-inflammatory events^17^. A major inflammatory mediator in GVHD is the NLRP3 inflammasome^18^ and caspase-1, the effector protease of the NLRP3 inflammasome, which is essential for pro-IL-1β maturation^19^.

We found in microarray-based studies that NLRP3 expression was upregulated in murine hematopoietic bone marrow (BM) cells harboring active inducible *Kras*^*G12D*^ allele. In agreement with a functional role of NLRP3 in the myeloid compartment, *Kras*^*G12D*^ BM-derived dendritic cells (BMDCs) showed increased IL-1β production and caspase-1 activation compared to wildtype (WT) cells. While mice expressing active Kras^G12D^ selectively in the hematopoietic system developed cytopenia and myeloproliferation, these disease features were abrogated in *Kras*^*G12D*^ mice lacking NLRP3 in the hematopoietic system. The findings in the mouse models could be recapitulated in patient samples of JMML, CMML, and AML patients carrying activating KRAS mutations. This study shows that oncogenic *Kras*^*G12D*^ leads to activation of the RAC1/ROS/NLRP3/IL-1β axis, which could be the basis for therapeutic approaches.

## Results

### Oncogenic Kras^G12D^ causes NLRP3 inflammasome and caspase-1 activation

**Fig. 1:**
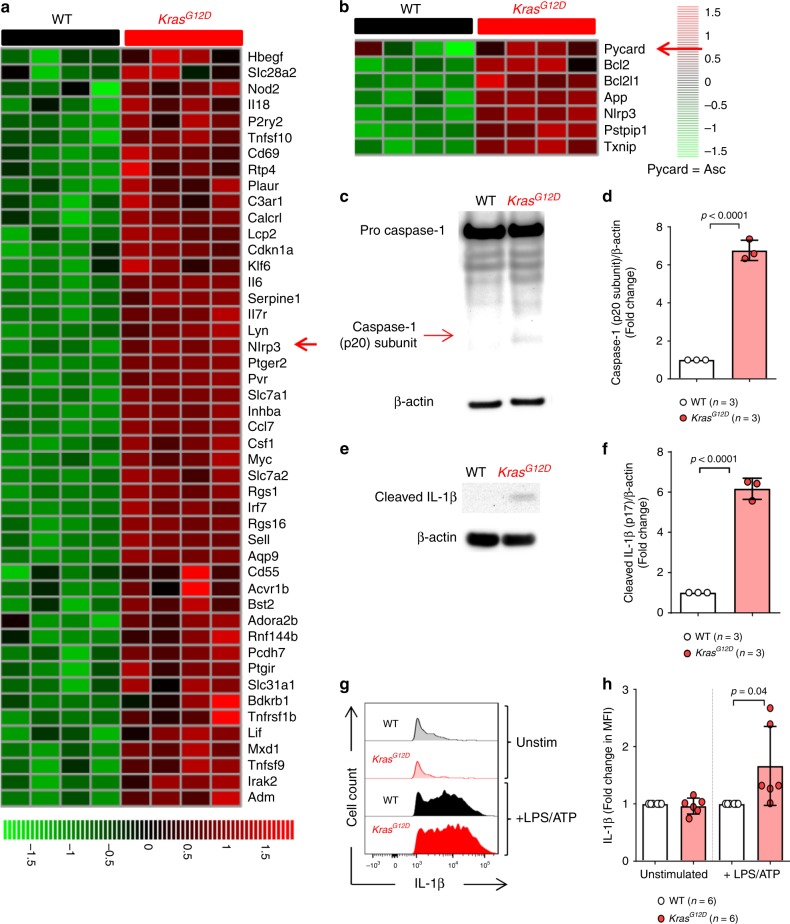
Oncogenic Kras^G12D^ leads to NLRP3 inflammasome activation in murine BM cells. **a** The heatmap represents the expression of inflammation-related genes in bone marrow-derived dendritic cells (BMDCs) isolated from either WT (*n* = 4) or *Kras*^*G12D*^ (*n* = 4) mice following treatment by tamoxifen. Color code represents the Z-score log2 intensity. **b** The heatmap represents the expression of inflammasomes upregulated genes from the Reactome gene set “inflammasome” in BMDCs isolated from either WT (*n* = 4) or *Kras*^*G12D*^ (*n* = 4) mice following treatment by tamoxifen. Color code represents the Z-score log2 intensity. **c** Western blot shows the amount of caspase-1 (p20 subunit) in WT or *Kras*^*G12D*^ BMDCs. The blot is representative for three independent experiments. **d** The ratio of caspase-1 (p20 subunit)/β-actin in WT (*n* = 3) or *Kras*^*G12D*^ (*n* = 3) BMDCs normalized to the WT. **e** Western blot shows the amount of cleaved IL-1β (p17) in WT or *Kras*^*G12D*^ BMDCs. The blot is representative for three independent experiments. **f** The ratio of cleaved IL-1β (p17)/ β-actin in WT (*n* = 3) or *Kras*^*G12D*^ (*n* = 3) BMDCs normalized to the WT. **g** The histogram shows mean fluorescence intensity (MFI) for IL-1β in WT or *Kras*^*G12D*^ BMDCs. One representative experiment from four experiments with a comparable pattern is shown. **h** The graph displays the fold change of IL-1β expression as measured by flow cytometry in WT (*n* = 6) or *Kras*^*G12D*^ (*n* = 6) BMDCs, normalized to the WT in both stimulation conditions. Pooled data of six biological replicates per group is shown. All data are shown as mean ± SEM.

To understand whether oncogenic Kras^G12D^ activates inflammation-related pathways, we used a conditional mouse model (*Rosa26Cre-*ER^T2^; *LSL-Kras*^*G12D*^) in which Kras^G12D^ can be induced upon tamoxifen adminstration, leading to Cre mediated recombination from its endogenous locus (Supplementary Fig. S1A, B). We studied, by microarray-based analysis, BM isolated from *Rosa26*Cre-ER^T2^; *LSL-Kras*^*G12D*^ mice or littermate controls after induction of Kras^G12D^ with tamoxifen. Clustering according to genes with the annotation “inflammation” divided WT versus *Kras*^*G12D*^ BM into two groups (Fig. 1a). Within the *Kras*^*G12D*^ BM, the *Nlrp3* gene was highly significant upregulated (Fig. 1a, red arrow), and a selective clustering of the gene set inflammasome from Reactome showed upregulation of multiple NLRP3 inflammasome related genes (Fig. 1b). In contrast to the NLRP3 inflammasome genes  *Nlrp3* and *Pycard* (Fig. 1b, red arrow), other inflammasome genes including *Casp1*, *GSDMD*, *Aim2*, *Nlrp1,* and *Nlrc4* were not upregulated in the *Kras*^*G12D*^ BM (Supplementary Fig. S1C). To test for activity of the NLRP3 inflammasome in *Kras*^*G12D*^ BM, we quantified caspase-1 auto-maturation in unprimed cells. In agreement with increased *Nlrp3* gene expression, highly enriched *Kras*^*G12D*^ BMDCs (Supplementary Fig. S1D) showed increased caspase-1 cleavage (p20 subunit detectable) compared to WT cells (Fig. 1c, d), as well as increased IL-1β cleavage (p17 detectable) (Fig. 1e, f), suggesting stronger inflammasome activation. Active caspase-1 mediates pro-IL-1β maturation into its bioactive form. IL-1β RNA transcription is initiated by TLR4/MyD88 signaling which can be induced by LPS^20^. Consistently, we observed increased amounts of IL-1β when *Kras*^*G12D*^ BMDCs were stimulated with lipopolysaccharide/adenosine-5′-triphosphate (LPS/ATP) compared to WT BMDCs (Fig. 1g, h). The IL-1β increase was not seen in the absence of LPS stimulation, which is in agreement with the requirement for TLR4/MyD88/TRIFF signaling for pro-IL-1β RNA transcription.

### NLRP3 deficiency reverses Kras^G12D^-induced cytopenia

**Fig. 2:**
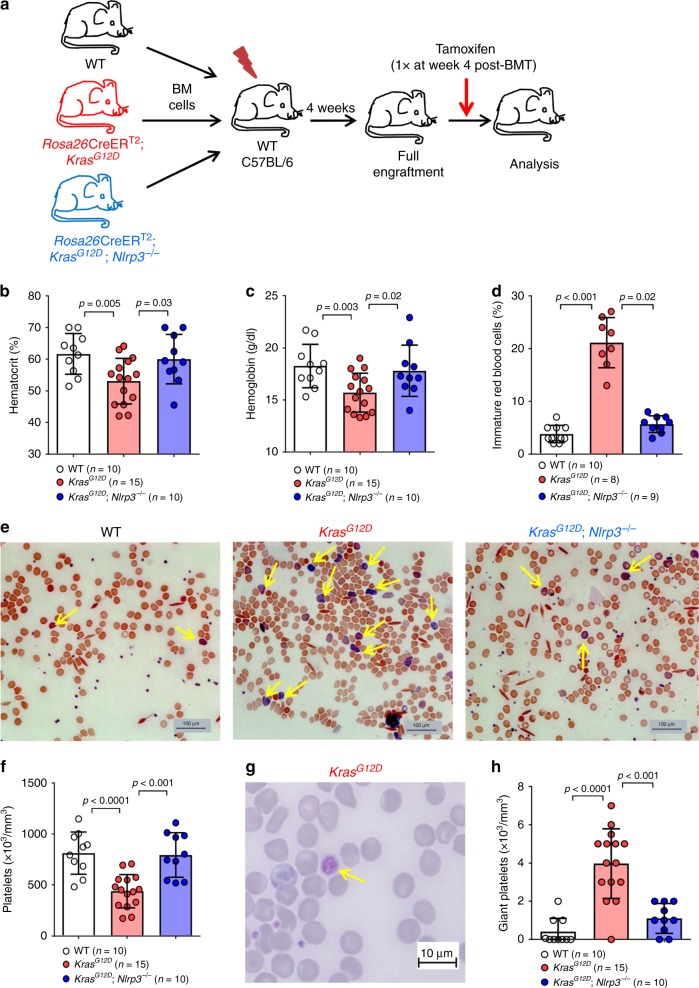
Absence of NLRP3 reverses the effects of *Kras*^*G12D*^ in peripheral blood. **a** Schematic diagram summarizing the experimental plan for generating BM chimeras that have WT BM, *Kras*^*G12D*^ BM or *Kras*^*G12D*^; *Nlrp3*^−/−^ BM. **b** The percentage of hematocrit in PB of WT (*n* = 10), *Kras*^*G12D*^ (*n* = 15) and *Kras*^*G12D*^; *Nlrp3*^−/−^ (*n* = 10) BM mice. **c** The concentrations of hemoglobin in PB of WT (*n* = 10), *Kras*^*G12D*^ (*n* = 15) and *Kras*^*G12D*^; *Nlrp3*^−/−^ (*n* = 10) BM mice. **d** The percentage of immature red blood cells observed in PB of WT (*n* = 10), *Kras*^*G12D*^ (*n* = 8) and *Kras*^*G12D*^; *Nlrp3*^−/−^ (*n* = 9) BM mice. **e** Representative PB smears images showing the increase in immature blood cells (marked with yellow arrows) in *Kras*^*G12D*^ BM mice, as compared to WT and *Kras*^*G12D*^; *Nlrp3*^−/−^ BM mice (Scale bar, 100 µm). The images are representative for three independent experiments. **f** Platelet counts in the PB of WT (*n* = 10), *Kras*^*G12D*^ (*n* = 15) and *Kras*^*G12D*^; *Nlrp3*^−/−^ (*n* = 10) BM mice are shown. **g** Representative PB smear image showing the enlarged size of platelets in *Kras*^*G12D*^ BM mice (Scale bar, 10 µm). **h** The number of giant platelets counted in PB smears of WT (*n* = 10), *Kras*^*G12D*^ (*n* = 15) and *Kras*^*G12D*^; *Nlrp3*^−/−^ (*n* = 10) BM mice. All data are shown as mean ± SEM.

To investigate a functional role of the NLRP3 inflammasome for the effects induced by oncogenic Kras^G12D^, we next generated mice with Kras^G12D^ activation and NLRP3 deficiency by crossing *Rosa26*Cre-ER^T2^; *LSL-Kras*^*G12D*^ mice onto a NLRP3-deficient background (*Nlrp3*^−/−^). The strain was termed *Rosa26*Cre-ER^T2^; *LSL-Kras*^*G12D*^; *Nlrp3*^−/−^ mice (*Kras*^*G12D*^; *Nlrp3*^−/−^). To avoid a possible indirect effect of active *Kras* in non-hematopoietic cells, we generated BM chimera that had either WT or *Kras*^*G12D*^ or *Kras*^*G12D*^ and *Nlrp3*^−/−^ in the hematopoietic compartment (Fig. 2a). Mice with *Kras*^*G12D*^ expression in hematopoietic system were termed *Kras*^*G12D*^ BM mice and mice with *Kras*^*G12D*^ and *Nlrp3*^−/−^ in the hematopoietic system were termed *Kras*^*G12D*^; *Nlrp3*^−/−^ BM mice. We found that the frequency of CD45 positive cells was equal in all three groups and we could show that the myeloablative conditioning followed by the BM transfer led to full engraftment (Supplementary Fig. S1E, F). We then administered tamoxifen to recipient mice to induce oncogenic Kras^G12D^ and later analyzed the phenotype in the peripheral blood (PB), BM and spleen. We observed an increase in lactate dehydrogenase (LDH) release in KrasG12D BM compared to WT BM, while there was no difference in LDH release in KrasG12D BM compared to KrasG12D; Nlrp3^−/−^ BM (Supplementary Fig. S1G). *Kras*^*G12D*^ BM mice developed anemia (decreased hemoglobin concentration and hematocrit) and an increase of reticulocytes (immature red blood cells) that were identified based on their higher size compared to mature erythrocytes and the scattered reticulum network in the cytoplasm which is visible as a blue granular precipitate^21^ (Fig. 2b–e). This phenotype was not seen in *Kras*^*G12D*^; *Nlrp3*^−/−^ BM mice (Fig. 2b–e). In addition, *Kras*^*G12D*^ BM mice developed low platelet counts and giant platelets were found in the peripheral blood and were not seen in *Kras*^*G12D*^; *Nlrp3*^−/−^ BM mice (Fig. 2f–h).

### NLRP3 absence counteracts Kras^G12D^ induced myeloproliferation

**Fig. 3:**
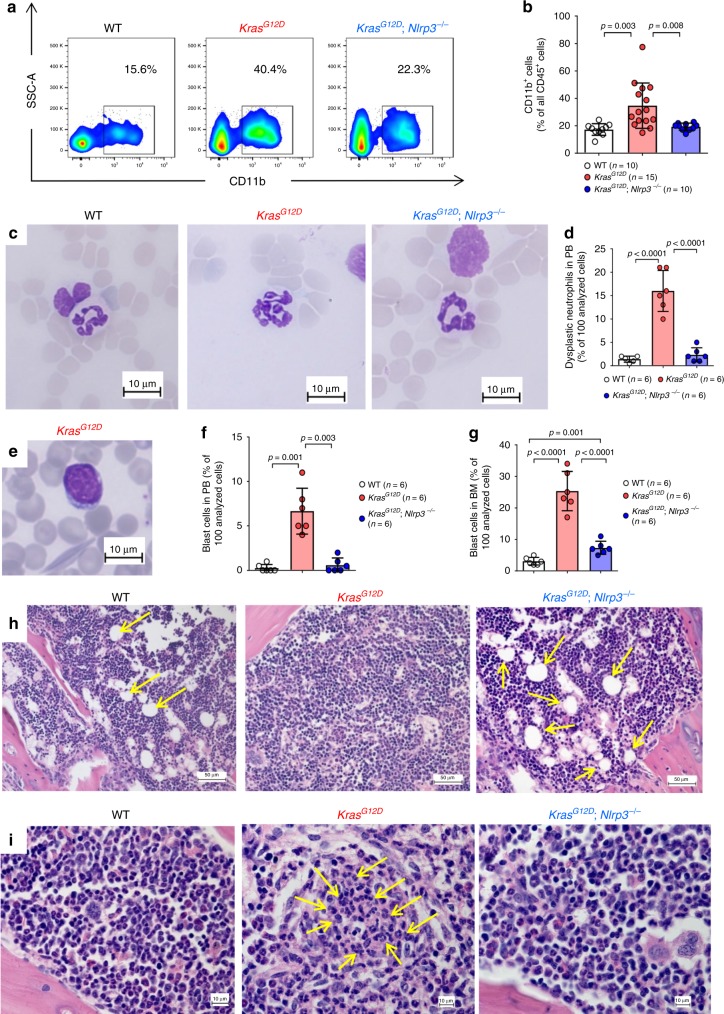
NLRP3 deficiency reverses myeloproliferation observed in *Kras*^*G12D*^ mice. **a** The plot shows the percentage of CD11b^+^ cells in PB of WT, *Kras*^*G12D*^ and *Kras*^*G12D*^; *Nlrp3*^−/−^ BM mice. One representative experiment from three experiments is shown. **b** The percentage of CD11b^+^ in PB of WT (*n* = 10), *Kras*^*G12D*^ (*n* = 15) and *Kras*^*G12D*^; *Nlrp3*^−/−^ (*n* = 10) BM mice is shown. **c** Representative PB smears images of WT, *Kras*^*G12D*^ and Kras^G12D^; *Nlrp3*^−/−^ BM mice showing neutrophil granulocytes with a dysplastic phenotype in *Kras*^*G12D*^ BM mice which is absent in WT and *Kras*^*G12D*^; *Nlrp3*^−/−^ BM mice (Scale bar, 10 µm).. Dysplasia diagnosis was based on hyperlobation of the nucleus. **d** The number of dysplastic neutrophils counted in PB isolated from WT (*n* = 6), *Kras*^*G12D*^ (*n* = 6) and *Kras*^*G12D*^; *Nlrp3*^−/−^ (*n* = 6) BM mice is shown. The counts represent percentage of 100 analyzed cells. **e** Representative PB smears image of a *Kras*^*G12D*^ mouse showing a blast which is absent in WT and *Kras*^*G12D*^; *Nlrp3*^−/−^ BM mice (Scale bar, 10 µm). The image is representative for three independent experiments. **f** The number of blast cells counted in PB isolated from WT (*n* = 6), *Kras*^*G12D*^ (*n* = 6) and *Kras*^*G12D*^; *Nlrp3*^−/−^ (*n* = 6) BM mice. The counts represent percentage of 100 analyzed cells. **g** The number of blast cells counted in BM isolated from WT (*n* = 6), *Kras*^*G12D*^ (*n* = 6) and *Kras*^*G12D*^; *Nlrp3*^−/−^ (*n* = 6) BM mice. The counts represent percentage of 100 analyzed cells. **h**, **i** Representative H&E stained BM images of WT, *Kras*^*G12D*^ and *Kras*^*G12D*^; *Nlrp3*^−/−^ BM mice showing (**h**) hypercellular BM with reduced fat vacuoles (marked with yellow arrows) (Scale bar, 50 µm). and (**i**) clusters of immature neutrophils (marked with yellow arrows) in *Kras*^*G12D*^ BM mice compared to normal BM phenotype observed in WT and *Kras*^*G12D*^; *Nlrp3*^−/−^ BM mice (Scale bar, 10 µm). The images are representative for three independent experiments. All data are shown as mean ± SEM.

We then analyzed the leukocytes in more detail and found increased frequencies of myeloid CD11b^+^ cells in the PB of *Kras*^*G12D*^ BM mice which were not seen in *Kras*^*G12D*^; *Nlrp3*^−/−^ BM mice (Fig. 3a, b). Neutrophil granulocytes with a dysplastic phenotype and blasts were more frequent in *Kras*^*G12D*^ BM mice compared to WT or *Kras*^*G12D*^; *Nlrp3*^−/−^ BM mice (Fig. 3c–f). Dysplasia was identified via the presence or absence of hyperlobation of the nucleus of neutrophils. Next, we asked whether NLRP3 deficiency would also influence other *Kras*^*G12D*^ mediated effects. We found increased amounts of blasts and promonocytes in the BM of *Kras*^*G12D*^ BM mice compared to WT or *Kras*^*G12D*^; *Nlrp3*^−/−^ BM mice (Fig. 3g). The number of blasts in the PB was increased in *Kras*^*G12D*^; *Nlrp3*^−/−^ BM mice compared to WT mice (Fig. 3g). When analyzing the BM morphology, *Kras*^*G12D*^ BM mice exhibited hypercellularity with reduced lipid-rich adipose cells and clusters of immature granulocytic cells (Fig. 3h, i).

**Fig. 4:**
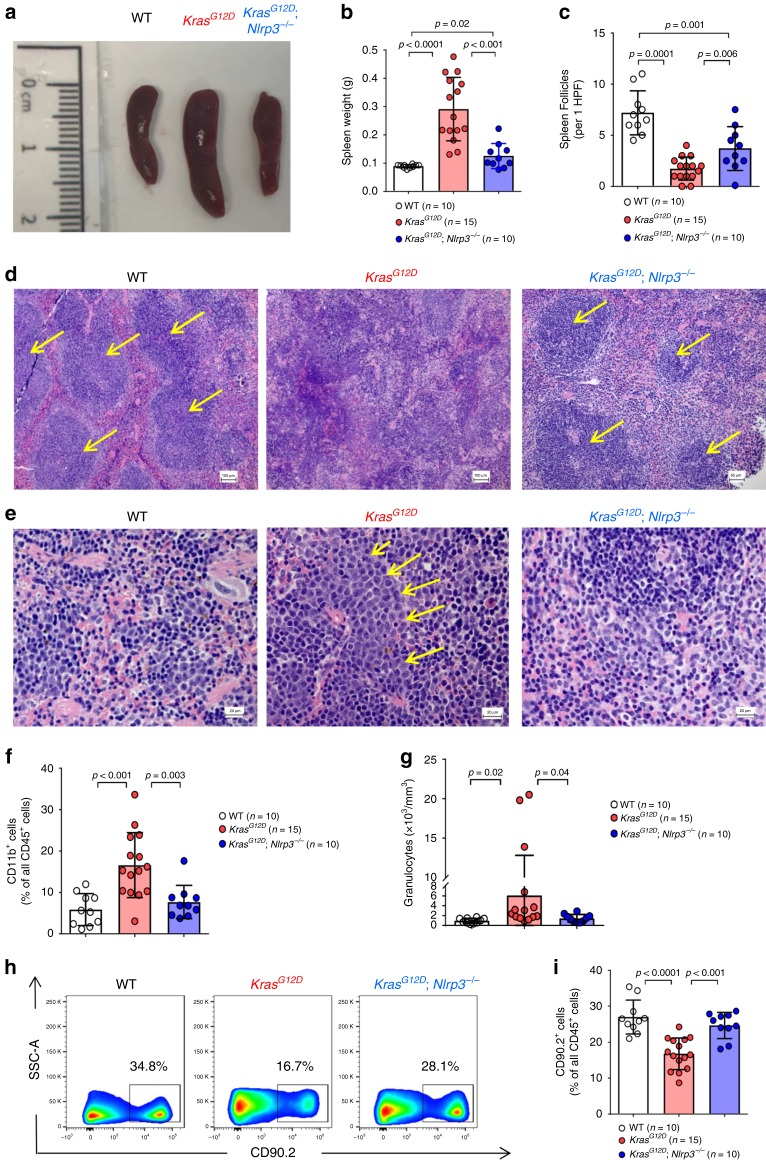
Kras^G12D^ induces splenomegaly which is reversed in *Kras*^*G12D*^; *Nlrp3*^*−/−*^ BM mice. **a** Representative image displaying spleen sizes of WT, *Kras*^*G12D*^ and *Kras*^*G12D*^; *Nlrp3*^−/−^ BM mice at the end of the experiment. **b** Measurements of spleen weight of WT (*n* = 10), *Kras*^*G12D*^ (*n* = 15) and *Kras*^*G12D*^; *Nlrp3*^−/−^ (*n* = 10) BM mice. **c** The number of spleen follicles counted in spleens isolated from WT (*n* = 10), *Kras*^*G12D*^ (*n* = 15) and *Kras*^*G12D*^; *Nlrp3*^−/−^ (*n* = 10) BM mice, per 1 high power field (HPF). **d**, **e** Photomicrographs of H&E stained splenic tissue sections of WT, *Kras*^*G12D*^ and *Kras*^*G12D*^; *Nlrp3*^−/−^ BM mice showing (**d**) disrupted splenic architecture in *Kras*^*G12D*^ (Scale bar, 100 µm) compared to WT (Scale bar, 100 µm) and *Kras*^*G12D*^; *Nlrp3*^−/−^ (Scale bar, 50 µm) BM mice which show typical normal follicles (marked with yellow arrows) with preserved mantle zones. (**e**) Abnormal infiltrates of myeloid progenitor cells predominantly in the splenic red pulp of *Kras*^*G12D*^ but not in *Kras*^*G12D*^; *Nlrp3*^−/−^ BM mice (Scale bar, 20 µm). **d** The yellow arrows indicate spleen follicles. **e** The yellow arrows indicate myeloid progenitor cells. **f** The percentage of CD11b^+^ cells in spleens of WT (*n* = 10), *Kras*^*G12D*^ (*n* = 15) and *Kras*^*G12D*^; *Nlrp3*^−/−^ (*n* = 10) BM mice. **g** Shown is the abundance of granulocytes in PB of WT (*n* = 10), *Kras*^*G12D*^ (*n* = 15), and *Kras*^*G12D*^; *Nlrp3*^−/−^ (*n* = 10) BM mice. **h** The plot shows the percentage of CD90.2^+^ in spleens of WT, *Kras*^*G12D*^ and *Kras*^*G12D*^; *Nlrp3*^−/−^ BM mice. One representative experiment from three experiments is shown. (**i**) The percentage of CD90.2^+^ in spleens of WT (*n* = 10), *Kras*^*G12D*^ (*n* = 15), and *Kras*^*G12D*^; *Nlrp3*^−/−^ (*n* = 10) BM mice. All data are shown as mean ± SEM.

Moreover, *Kras*^*G12D*^ BM mice developed splenomegaly, which was not seen in *Kras*^*G12D*^; *Nlrp3*^−/−^ BM mice (Fig. 4a, b). In agreement with this finding, splenomegaly is a disease feature of JMML driven by *KRAS* activation^22^. In contrast to the spleen, organs that do not belong to the hematopoietic/lymphatic system (e.g. kidney, heart) were not enlarged indicating that the effect of *KRAS* activation was restricted to the hematopoietic/lymphatic system. Also, the splenic architecture comprising white pulp lymphoid follicles with germinal centers and marginal zone areas in WT mice was disrupted in *Kras*^*G12D*^ BM mice but not in *Kras*^*G12D*^; *Nlrp3*^−/−^ BM mice (Fig. 4c, d). We found abnormal infiltrates of myeloid progenitor cells in the spleens of *Kras*^*G12D*^ BM mice but not in *Kras*^*G12D*^; *Nlrp3*^−/−^ BM mice (Fig. 4e). Moreover, myeloid CD11b^+^ cells were increased in the spleens of *Kras*^*G12D*^ BM mice but not in *Kras*^*G12D*^; *Nlrp3*^−/−^ BM mice (Fig. 4f). Consistent with these findings in the spleen and BM, we observed higher numbers of granulocytes in the PB of *Kras*^*G12D*^ BM mice but not in *Kras*^*G12D*^; *Nlrp3*^−/−^ BM mice (Fig. 4g). Conversely, the percentages of T cells of all CD45^+^ cells were decreased in *Kras*^*G12D*^ BM mice (Fig. 4h, i).

**Fig. 5:**
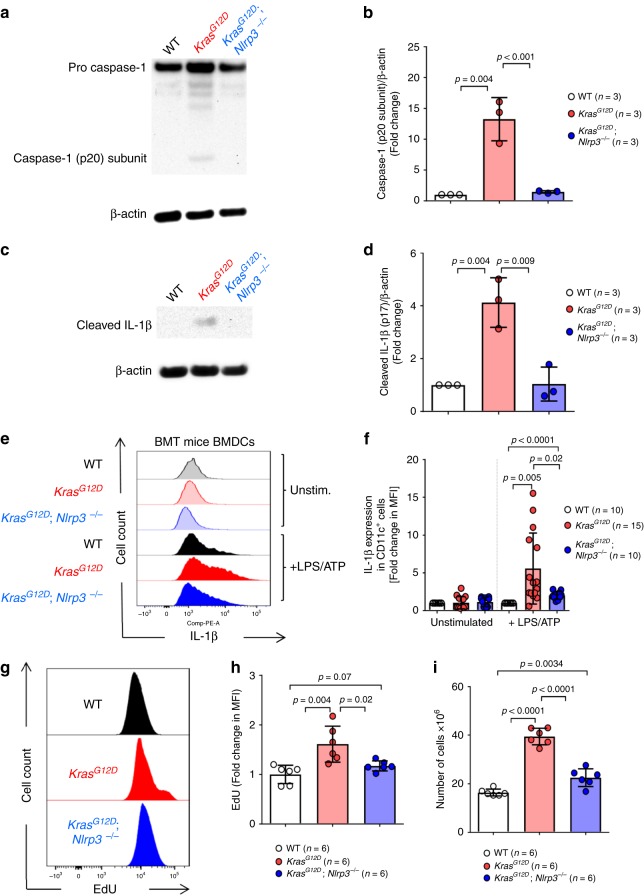
Increased NLRP3 inflammasome activation by Kras^G12D^ is reversed the absence of NLRP3. **a** Western blot shows the amount of caspase-1 (p20 subunit) in BMDCs generated from WT, *Kras*^*G12D*^ and *Kras*^*G12D*^; *Nlrp3*^−/−^ BM mice. The blot is representative for three independent experiments. **b** The ratio of caspase-1 (p20 subunit)/β-actin in BMDCs generated from WT (*n* = 3), *Kras*^*G12D*^ (*n* = 3), and *Kras*^*G12D*^; *Nlrp3*^−/−^ (*n* = 3) BM mice, normalized to WT. **c** Western blot shows the amount of cleaved IL-1β (p17) in BMDCs generated from WT, *Kras*^*G12D*^ and *Kras*^*G12D*^; *Nlrp3*^−/−^ BM mice. The blot is representative for three independent experiments. **d** The ratio of cleaved IL-1β (p17)/β-actin in BMDCs generated from WT (*n* = 3), *Kras*^*G12D*^ (*n* = 3) and *Kras*^*G12D*^; *Nlrp3*^−/−^ (*n* = 3) BM mice, normalized to WT. **e** The histogram shows mean fluorescence intensity (MFI) for IL-1β in BMDCs generated from WT, *Kras*^*G12D*^ and *Kras*^*G12D*^; *Nlrp3*^−/−^ BM mice. One representative experiment from three experiments is shown. **f** The graph displays the fold change of IL-1β expression as measured by flow cytometry in BMDCs generated from WT (*n* = 10), *Kras*^*G12D*^ (*n* = 15) and *Kras*^*G12D*^; *Nlrp3*^−/−^ (*n* = 10) BM mice, normalized to the WT in both stimulation conditions. **g** The histogram shows mean fluorescence intensity (MFI) for EdU in BMDCs generated from WT, *Kras*^*G12D*^ or *Kras*^*G12D*^; *Nlrp3*^−/−^ mice. One representative experiment from two experiments is shown. **h** The graph displays the fold change of cell proliferation measured by EdU incorporation in BMDCs generated from WT (*n* = 6), *Kras*^*G12D*^ (*n* = 6), or *Kras*^*G12D*^; *Nlrp3*^−/−^ (*n* = 6) mice, normalized to the WT. **i** The graph displays the number of cells counted following BMDCs generation using isolated BM from WT (*n* = 6), *Kras*^*G12D*^ (*n* = 6), or *Kras*^*G12D*^; *Nlrp3*^−/−^ (*n* = 6) mice. All data are shown as mean ± SEM.

To clarify whether the NLRP3 inflammasome was active in myeloid cells of *Kras*^*G12D*^ BM mice, we studied cleaved caspase-1 and cleaved IL-1β (p17 detectable) concentrations in BMDCs (enrichment purity: Supplementary Fig. S2A). In agreement with increased NLRP3 inflammasome activation, *Kras*^*G12D*^ BMDCs led to increased cleaved caspase-1 (Fig. 5a, b), increased cleaved IL-1β (p17 detectable) in cell lysates (Fig. 5c, d) and in cell-free supernatants (Supplementary Fig. S2B), compared to WT and *Kras*^*G12D*^; *Nlrp3*^−/−^ BMDCs. Additionally, we observed increased amounts of IL-1β in *Kras*^*G12D*^ BMDCs compared to WT and *Kras*^*G12D*^; *Nlrp3*^−/−^ BMDCs (Fig. 5e, f, Supplementary Fig. S2C), following stimulation. The differences observed between WT and *Kras*^*G12D*^; *Nlrp3*^−/−^ BMDCs indicate that *KRAS* does not exclusively cause the phenotype by IL-1β release, but also by a direct pro-proliferative effect. The IL-1β increase was not seen in the absence of LPS stimulation, which is in agreement with the need for TLR4/MyD88/TRIFF signaling for IL-1β RNA transcription.

To evaluate whether the connection between oncogenic Kras^G12D^ and NLRP3 is cell-autonomous, we analyzed if the myeloproliferation caused by the KRAS/NLRP3 axis in vivo would also be active in vitro in the absence of a microenvironment. We observed that proliferation of myeloid cells in vitro was higher when the cells were derived from *Kras*^*G12D*^ mice compared to WT mice (Fig. 5g–i). We also found that proliferation in BMDCs generated from *Kras*^*G12D*^ was higher compared to BMDCs generated from *Kras*^*G12D*^; *Nlrp3*^−/−^ mice indicating that NLRP3 deficiency reverses the Kras-induced phenotype (Fig. 5i). However, proliferation in BMDCs generated from *Kras*^*G12D*^; *Nlrp3*^−/−^ mice was higher compared to BMDCs generated from WT mice (Fig. 5i). These findings support the concept that the connection between oncogenic Kras^G12D^ and NLRP3 is partly cell-autonomous and partly via impacting the microenvironment. An immunosuppressive, pro-tumorigenic role of IL-1β in the tumor microenvironment has been reported before^23^.

### NLRP3 inhibition reverses myeloproliferation by *Kras*^*G12D*^

**Fig. 6:**
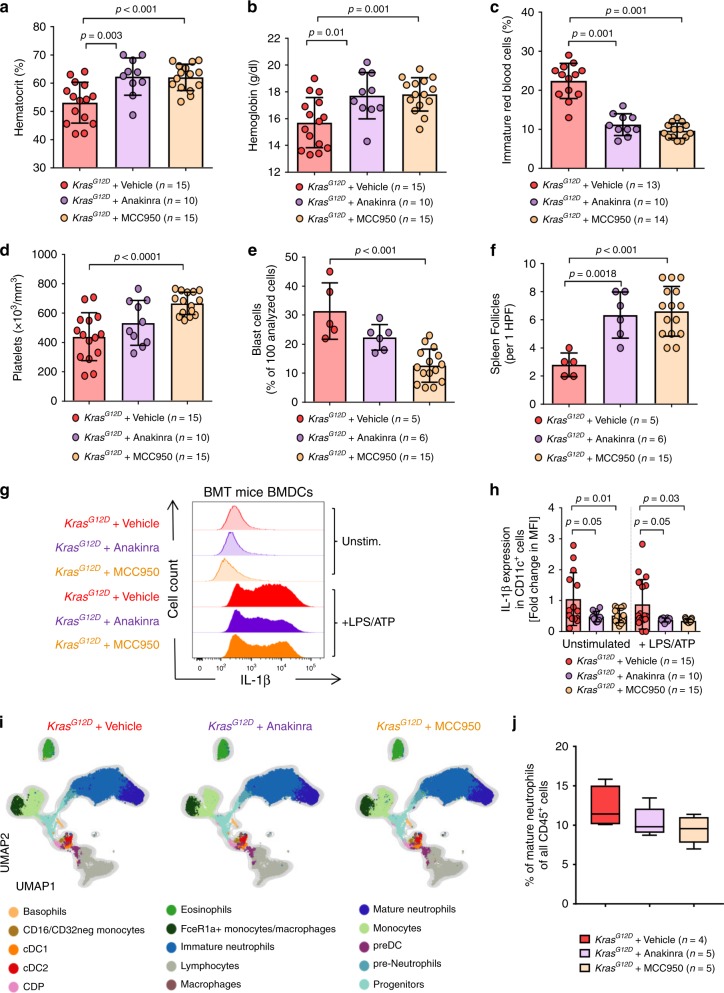
IL-1R- or NLRP3- inhibition in *Kras*^*G12D*^ mice leads to reduced myeloproliferation. **a** The percentage of hematocrit in PB of vehicle-treated *Kras*^*G12D*^ (*n* = 15), Anakinra-treated *Kras*^*G12D*^ (*n* = 10) or MCC950-treated *Kras*^*G12D*^ (*n* = 15) BM mice. Both Anakinra and NLRP3 inhibitor (MCC950) treatment started 3 weeks post-tamoxifen and ended a day before sacrificing the mice (treatment was in total for 4 weeks). **b** The concentrations of hemoglobin in PB of vehicle-treated *Kras*^*G12D*^ (*n* = 15), Anakinra-treated *Kras*^*G12D*^ (*n* = 10) or MCC950-treated *Kras*^*G12D*^ (*n* = 15) BM mice. **c** The percentage of immature red blood cells observed in PB of vehicle-treated *Kras*^*G12D*^ (*n* = 15), Anakinra-treated *Kras*^*G12D*^ (*n* = 10) or MCC950-treated *Kras*^*G12D*^ (*n* = 15) BM mice. **d** Shown are the number of platelets in PB of vehicle-treated *Kras*^*G12D*^ (*n* = 15), Anakinra-treated *Kras*^*G12D*^ (*n* = 10) or MCC950-treated *Kras*^*G12D*^ (*n* = 15) BM mice. **e** The number of blast cells counted in BM isolated from vehicle-treated *Kras*^*G12D*^ (*n* = 5), Anakinra-treated *Kras*^*G12D*^ (*n* = 6) or MCC950-treated *Kras*^*G12D*^ (*n* = 15) BM mice. The counts represent percentage of 100 analyzed cells. **f** The number of spleen follicles counted in spleens isolated from vehicle-treated *Kras*^*G12D*^ (*n* = 5), Anakinra-treated *Kras*^*G12D*^ (*n* = 6) or MCC950-treated *Kras*^*G12D*^ (*n* = 15) BM mice, per 1 high power field (HPF). **g** The histogram shows mean fluorescence intensity (MFI) for IL-1β in BMDCs of vehicle-treated *Kras*^*G12D*^, Anakinra-treated *Kras*^*G12D*^ or MCC950-treated *Kras*^*G12D*^ BM mice. One representative experiment from three experiments is shown. **h** The graph displays the fold change of IL-1β expression as measured by flow cytometry in BMDCs of vehicle-treated *Kras*^*G12D*^ (*n* = 15), Anakinra-treated *Kras*^*G12D*^ (*n* = 10) or MCC950-treated *Kras*^*G12D*^ (*n* = 15) BM mice, normalized to vehicle-treated *Kras*^*G12D*^ in both stimulation conditions. **i** FlowSOM-guided metaclustering of CD45^+^ BM cells (gated on live/single cells/CD45^+^) per condition: vehicle-treated *Kras*^*G12D*^ (*n* = 4), Anakinra-treated *Kras*^*G12D*^ (*n* = 5) or MCC950-treated *Kras*^*G12D*^ (*n* = 5) BM mice. **j** Frequency of FlowSOM-guided mature neutrophils cluster among CD45^+^ BM cells in vehicle-treated *Kras*^*G12D*^ (*n* = 4), Anakinra-treated *Kras*^*G12D*^ (*n* = 5) or MCC950-treated *Kras*^*G12D*^ (*n* = 5) BM mice. All data are shown as mean ± SEM.

To study the functional role of the NLRP3/IL-1β axis, we treated *Kras*^*G12D*^ BM mice with the IL-1 receptor type 1 antagonist Anakinra (recombinant IL-1RA) or the NLRP3 inhibitor MCC950 for 4 weeks, starting 3 weeks post-tamoxifen when the disease was established until the day before analysis. We observed increased hematocrit and hemoglobin concentrations in *Kras*^*G12D*^ BM mice treated with Anakinra or MCC950 (Fig. 6a, b), compared to *Kras*^*G12D*^ BM mice treated with vehicle. In addition, there was a decrease in the percentage of immature red blood cells in PB of Anakinra-treated *Kras*^*G12D*^ BM mice, compared to vehicle-treated *Kras*^*G12D*^ BM mice (Fig. 6c). Platelet counts increased in *Kras*^*G12D*^ BM mice treated with Anakinra or MCC950 (Fig. 6d). Moreover, Anakinra or MCC950 treatment could reverse the spleen and BM phenotype in *Kras*^*G12D*^ recipient mice (Fig. 6e, f). Consistent with a reduced KRAS/NLRP3/IL-1β axis, we observed decreased amounts of IL-1β in BMDCs (enrichment purity: Supplementary Fig. S2D) generated from Anakinra-treated or MCC950-treated *Kras*^*G12D*^ BM mice compared to vehicle-treated *Kras*^*G12D*^ BM mice (Fig. 6g, h). Importantly the inhibitor treatment did not lead to a depletion of a certain cell type (Fig. 6i, Supplementary Fig. S3A, B) but only to a mild reduction of mature neutrophils that did not reach significance (Fig. 6j). These findings indicate that NLRP3 and IL-1R are therapeutic targets to interfere with *KRAS*-driven myeloproliferation.

### Myeloproliferation is mediated via the RAC1/ROS/NLRP3 axis

**Fig. 7:**
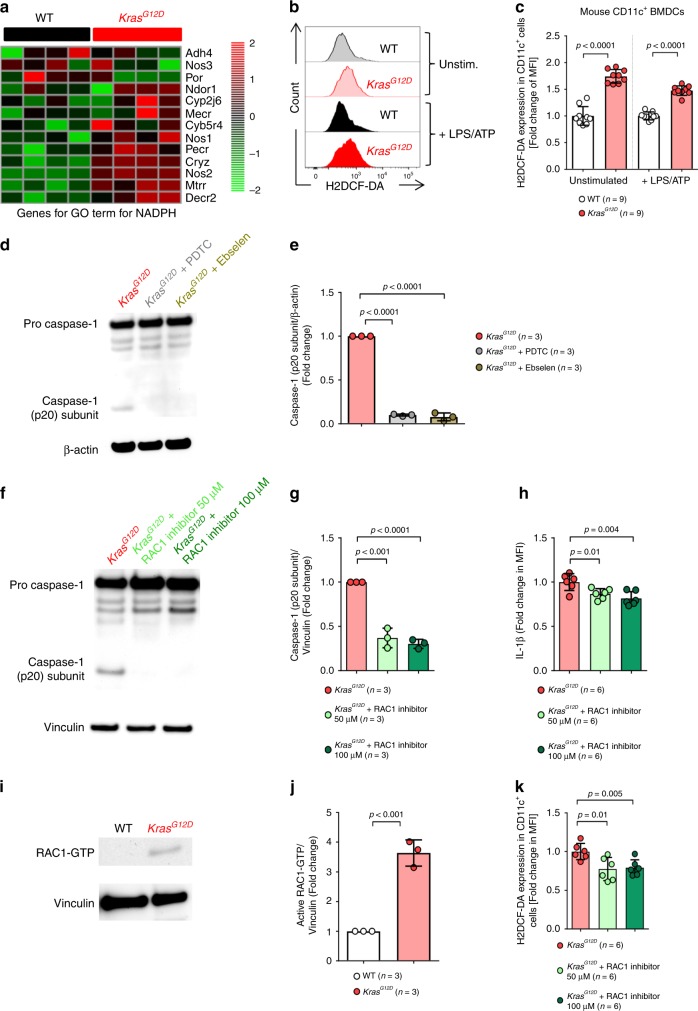
Kras^G12D^ causes inflammasome activation RAC1 and reactive oxygen species (ROS) production. **a** The heatmap shows genes for upregulated GO terms for NADPH in BMDCs generated from either WT (*n* = 4) or *Kras*^*G12D*^ (*n* = 4) mice following treatment by tamoxifen. Color code represents the Z-score log2 intensity. **b** The histogram shows mean fluorescence intensity (MFI) for H2DCF-DA in WT or *Kras*^*G12D*^ BMDCs. **c** The graph displays the fold change of H2DCF-DA expression as measured by flow cytometry in WT (*n* = 9) and *Kras*^*G12D*^ (*n* = 9) BMDCs, normalized to WT in both stimulation conditions. **d** Western blot shows the amount of caspase-1 (p20 subunit) in BMDCs isolated from *Kras*^*G12D*^ mice treated with DMSO, Ebselen or PDTC, after stimulation with 200 ng/ml LPS and 5 mM ATP. The blot is representative for two independent experiments. **e** The ratio of caspase-1 (p20 subunit)/β-actin in *Kras*^*G12D*^ BMDCs treated with DMSO (*n* = 3), Ebselen (*n* = 3) or PDTC (*n* = 3), normalized to DMSO-treated *Kras*^*G12D*^ BMDCs. **f** Western blot shows the amount of caspase-1 (p20 subunit) in BMDCs generated from *Kras*^*G12D*^ mice treated with DMSO, 50 µM or 100 µM of RAC1 inhibitor NSC 23766, after stimulation with 200 ng/ml LPS and 5 mM ATP, normalized to DMSO-treated *Kras*^*G12D*^ BMDCs. The blot is representative for two independent experiments. **g** The ratio of caspase-1 (p20 subunit)/Vinculin in *Kras*^*G12D*^ BMDCs treated with DMSO (*n* = 3), 50 µM (*n* = 3) or 100 µM (*n* = 3) of RAC1 inhibitor NSC 23766, normalized to DMSO-treated *Kras*^*G12D*^ BMDCs. **h** The graph displays the fold change of IL-1β expression as measured by flow cytometry in *Kras*^*G12D*^ BMDCs treated with DMSO (*n* = 6), 50 µM (*n* = 6) or 100 µM (*n* = 6) of RAC1 inhibitor NSC 23766, after stimulation with 200 ng/ml LPS and 5 mM ATP, normalized to DMSO-treated *Kras*^*G12D*^ BMDCs. **i** Western blot shows the amount of active RAC1-GTP in BMDCs generated from WT or *Kras*^*G12D*^ mice. **j** The ratio of active RAC1-GTP/Vinculin in BMDCs generated from WT (*n* = 3) or *Kras*^*G12D*^ (*n* = 3) BM, normalized to WT. **k** The graph displays the fold change of H2DCF-DA expression in BMDCs generated from *Kras*^*G12D*^ mice, treated with DMSO (*n* = 6), 50 µM (*n* = 6) or 100 µM (*n* = 6), normalized to DMSO-treated *Kras*^*G12D*^ BMDCs. All data are shown as mean ± SEM.

To understand the mechanism by which oncogenic *KRAS* leads to NLRP3 activation, we performed an unbiased gene expression analysis of WT or *Kras*^*G12D*^ BMDCs. We observed increased expression of genes for Gene Ontology (GO) terms related to NADPH oxidase, an enzyme required for the production of extracellular/endosomal ROS (Fig. 7a). In agreement, we found increased cellular ROS production in *Kras*^*G12D*^ BMDCs compared to WT (Fig. 7b, c). Blocking ROS production by PDTC or Ebselen (enrichment purity and viability: Supplementary Fig. S4A, B) abrogated the increased caspase-1 cleavage seen in *Kras*^*G12D*^ BMDCs (Fig. 7d, e).

To clarify which KRAS downstream signals activate NADPH and NLRP3, we next used MEK-specific inhibitors, an ERK inhibitor, PI3K-specific inhibitors and a RAC1 inhibitor and then studied ROS production and NLRP3 inflammasome activation. At the concentrations used the cell viability was not affected (Supplementary Fig. S4C). We observed no change in IL-1β expression in *Kras*^*G12D*^ BMDCs when MEK, ERK or PI3K were blocked by Trametinib, Selumetinib, Ulixertinib, Buparlisib and Pictilisib (Supplementary Fig. S4D). Conversely, caspase-1 cleavage and IL-1β expression were reduced in *Kras*^*G12D*^ BMDCs when RAC1 was blocked (Fig. 7f–h). Consistent with a role for RAC1, we observed increased levels of active RAC1-GTP in *Kras*^*G12D*^ BMDCs (Fig. 7i, j**)**. RAC1 inhibition, but not MEK, ERK or PI3K-inhibition reduced ROS production by *Kras*^*G12D*^ BMDCs (Fig. 7k, Supplementary Fig. S4E).

### NLRP3 inflammasome activation in human *KRAS*-mutant (Kras^mut^) leukemia cells

**Fig. 8:**
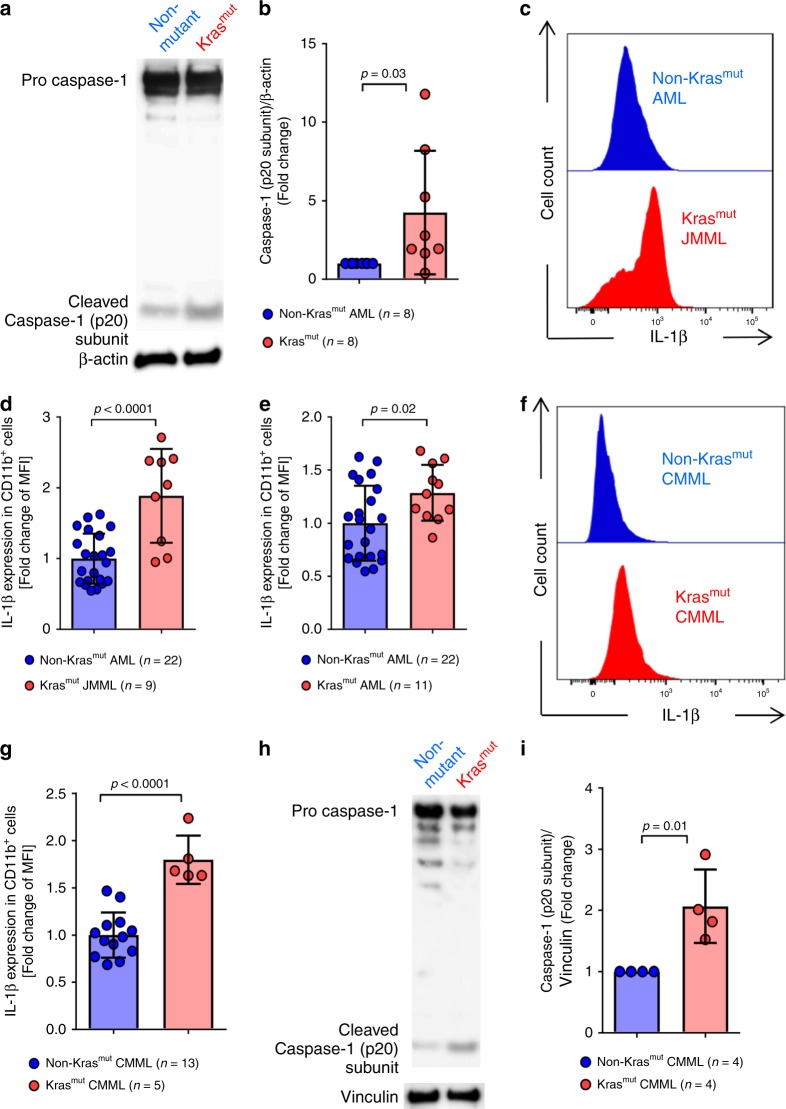
*KRAS*-mutant human leukemia cells exhibit increased NLRP3 inflammasome activation. **a** Western blot shows the amount of caspase-1 (p20 subunit) in PBMCs isolated from patients with a *KRAS* mutation (Kras^mut^) or without a *KRAS* mutation (non-Kras^mut^). The blot is representative for four independent experiments. **b** The ratio of caspase-1 (p20 subunit)/β-actin in Kras^mut^ (*n* = 8) or non-Kras^mut^ (*n* = 8) patients’ PBMCs normalized to non-Kras^mut^. **c** The histogram shows mean fluorescence intensity (MFI) for IL-1β in CD11b^+^ cells of Kras^mut^ JMML or non- Kras^mut^ AML patients. One representative experiment from four experiments with is shown. **d**–**e** The graphs display the fold change of IL-1β expression as measured by flow cytometry in (**d**) Kras^mut^ JMML (*n* = 9) and (**e**) Kras^mut^ AML (*n* = 11), compared to non-Kras^mut^ AML patients’ PBMCs (n = 22), normalized to non-Kras^mut^. (**f**) The histogram shows mean fluorescence intensity (MFI) for IL-1β in CD11b^+^ cells of Kras^mut^ or non-Kras^mut^ CMML patients’ cells. One representative experiment from four experiments is shown. (**g**) The graph displays MFI fold change of IL-1β expression as measured by flow cytometry in Kras^mut^ (*n* = 5) and non-Kras^mut^ (*n* = 13) CMML patients’ PBMCs, normalized to non-Kras^mut^. **h** Western blot shows the amount of caspase-1 (p20 subunit) in PBMCs isolated from CMML patients with a *KRAS* mutation (Kras^mut^) or without a *KRAS* mutation (non-Kras^mut^). **i** The ratio of caspase-1 (p20 subunit)/Vinculin in Kras^mut^ (*n* = 4) or non-Kras^mut^ (*n* = 4) CMML patients’ PBMCs normalized to non-Kras^mut^.

To determine whether the observations made in mouse cells were also seen in human cells, we isolated PBMCs of AML, JMML or CMML patients with *KRAS* mutations (Kras^mut^) and gated on CD11b^+^ cells (Supplementary Fig. S5A). In contrast to AML patients without *KRAS* mutations (Non-Kras^mut^), we observed increased cleaved caspase-1 in Kras^mut^ PBMCs (Fig. 8a, b), indicating increased NLRP3 inflammasome activity in human Kras^mut^ cells compared to non-mutant cells. In agreement with inflammasome activation, we also observed increased IL-1β production in human Kras^mut^ cells compared to non-mutant cells in JMML, AML, and CMML (Fig. 8c–g). The cell number and phenotype was not affected by the stimulation with LPS (Supplementary Fig. S5B).

To determine if the *KRAS* mutations were found in CD11b positive cells, we sorted for these cells, and analyzed the resulting DNA using targeted Next-Generation Sequencing (NGS) and digital droplet PCR (ddPCR) for the respective *KRAS* mutations. We could show that the *KRAS* mutations were found in CD11b^+^ sorted cells at a high variant allele frequency (VAF; 45%, 49% in representative samples, Supplementary Fig. S5C-E). In addition, we found increased cleaved caspase-1 in human Kras^mut^ CMML cells compared to non-Kras^mut^ cells (Fig. 8h, i). CMML samples with NRAS mutations (Nras^mut^) displayed only a small increase of IL-1β (Supplementary Fig. S5F).

### Increased ROS production in human Kras^mut^ myeloid leukemia cells

**Fig. 9:**
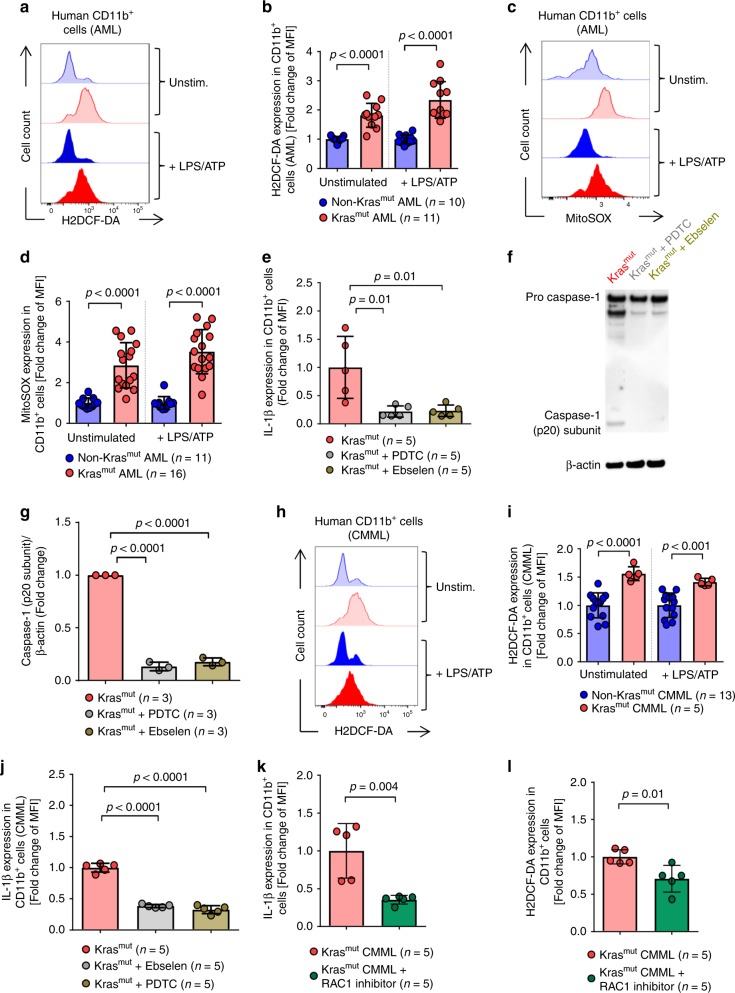
The *KRAS*/RAC1/ROS/NLRP3 axis is active in human leukemia cells. **a** The histogram shows mean fluorescence intensity (MFI) for H2DCF-DA in CD11b^+^ cells of AML patients with a *KRAS* mutation (Kras^mut^) or without a *KRAS* mutation (non-Kras^mut^). **b** The graph displays MFI fold change of H2DCF-DA expression in Kras^mut^ (*n* = 11) and non-Kras^mut^ (*n* = 10) AML patient cells, normalized to non-Kras^mut^ in both stimulation conditions. **c** The histogram shows MFI for MitoSOX in Kras^mut^ or non-Kras^mut^ AML patients cells. **d** The graph displays MFI fold change of MitoSOX expression in Kras^mut^ (*n* = 16) and non-Kras^mut^ (*n* = 11) patient cells, normalized to non-Kras^mut^ in both stimulation conditions. **e** The graph displays MFI fold change of IL-1β expression in Kras^mut^ JMML patients cells, treated with DMSO (*n* = 5), Ebselen (*n* = 5) or PDTC (*n* = 5), normalized to DMSO-treated Kras^mut^. **f** Western blot shows the amount of caspase-1 (p20 subunit) in PBMCs isolated from Kras^mut^ JMML patients treated with DMSO, Ebselen or PDTC after stimulation with 200 ng/ml LPS and 5 mM ATP. **g** The ratio of caspase-1 (p20 subunit)/β-actin in Kras^mut^ PBMCs derived from JMML patients, treated with DMSO (*n* = 3), Ebselen (*n* = 3) or PDTC (*n* = 3), normalized to DMSO-treated Kras^mut^ cells. **h** The histogram shows MFI for H2DCF-DA in Kras^mut^ or non-Kras^mut^ CMML patients cells. **i** The graph displays MFI fold change of H2DCF-DA expression in Kras^mut^ (*n* = 5) and non-Kras^mut^ (*n* = 13) CMML patients cells, normalized to non-Kras^mut^ in both stimulation conditions. **j** The graph displays MFI fold change of IL-1β expression in Kras^mut^ CMML patients cells, treated with DMSO (*n* = 5), Ebselen (*n* = 5) or PDTC (*n* = 5), normalized to DMSO-treated Kras^mut^ cells. **k** The graph displays MFI fold change of IL-1β expression in Kras^mut^ CMML patients cells, treated with DMSO (*n* = 5) or 100 µM of RAC1 inhibitor NSC 23766 (*n* = 5), after stimulation with 200 ng/ml LPS and 5 mM ATP, normalized to DMSO-treated Kras^mut^. **l** The graph displays MFI fold change of H2DCF-DA expression in Kras^mut^ CMML patients cells, treated with DMSO (*n* = 5) or 100 µM of RAC1 inhibitor NSC 23766 (*n* = 5), after stimulation with 200 ng/ml LPS and 5 mM ATP, normalized to DMSO-treated Kras^mut^. All data are shown as mean ± SEM.

Comparable to the observations made in mice, we found that ROS production was increased in human Kras^mut^ cells compared to non-Kras^mut^ cells derived from AML patients in the cytoplasm (Fig. 9a, b) and in the mitochondria (Fig. 9c, d). Blocking ROS production by PDTC or Ebselen reduced IL-1β production (Fig. 9e), and also abrogated the increased caspase-1 cleavage seen in Kras^mut^ JMML PBMCs (Fig. 9f, g). Increased ROS production was also seen in Kras^mut^ cells, but not Nras^mut^ cells, from CMML patients compared to non-Kras^mut^ CMML cells (Fig. 9h, i, Supplementary Fig. S5G). Blocking ROS production by PDTC or Ebselen reduced IL-1β production in CMML patient Kras^mut^ PBMCs (Fig. 9j). In addition, and similar to our findings in murine models, blocking RAC1 in Kras^mut^ cells, but not Nras^mut^ cells, from CMML patients caused a reduction of IL-1β expression and ROS production **(**Fig. 9k, l, Supplementary Fig. S5H, I**)**.

These observations in patient samples show that the KRAS/ROS/NLRP3/IL-1β axis was also active in human AML, CMML and JMML cells, which is in agreement with our observations in mouse models.

**Fig. 10:**
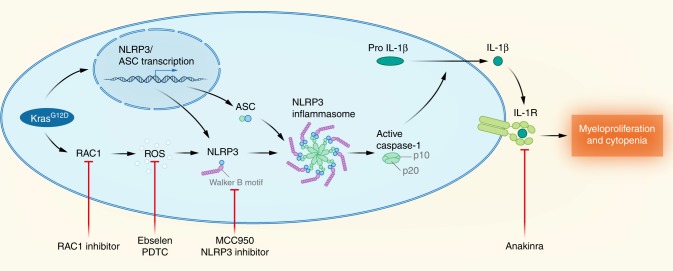
Proposed model. The scheme shows the proposed mechanism. Oncogenic *KRAS* leads to NLRP3/ASC transcription and RAC1-mediated production of reactive oxygen species (ROS). This licenses the NLRP3 inflammasome and activates the inflammasome by ROS leading to caspase-1 activation, ASC recruitment and consecutively to the release of bioactive IL-1β. The KRAS/NLRP3/IL-1β axis can be inhibited by blocking IL-1R or by inhibiting the NLRP3 inflammasome.

Our findings indicate that oncogenic *KRAS* leads to RAC1 activation, causing NADPH activation, resulting in ROS production, which in turn activates the NLRP3 inflammasome causing cleavage of caspase-1 and IL-1β production (Fig. 10; *proposed model*).

## Discussion

Improving the treatment of JMML, CMML, and KRAS-mutant AML is still a major unmet clinical need. Conventional chemotherapy in JMML has little effect^24^ and the probability of event-free survival at 5 years is only 50% after allogeneic hematopoietic cell transplantation (allo-HCT)^25^. Similarly, allo-HCT remains the only curative option for patients with CMML, with an overall survival at 3 years of 32%^12^. A major clinical feature of patients with JMML are inflammatory symptoms including hepatosplenomegaly in greater than 90%, lymphadenopathy in 76%, pallor in 64%, fever in 54%, skin rash in 36%^22,26^. Also in CMML hepatomegaly, splenomegaly and features of myeloproliferation, such as fatigue, symptoms from organomegaly, night sweats, weight loss and cachexia have been reported^12^. The pathomechanism causing these symptoms in KRAS-mutant myeloid neoplasms was not fully elucidated. Here we report that multiple inflammation-related genes are upregulated upon oncogenic *KRAS* activation in myeloid cells. A major inflammatory pathway we identified was the NLRP3/caspase-1/IL-1β axis. This is consistent with previous reports showing that IL-1β was increased in JMML^27,28^. These reports, however, did not clarify the mechanism underlying the increased IL-1β production, because the role of the inflammasome for IL-1β activity was unknown at this time. Our work identifies a critical role of the NLRP3 inflammasome in Kras^G12D^ induced inflammation, as the hematological phenotype caused by oncogenic *Kras*^*G12D*^ expression is reversed by NLRP3 deficiency. Our studies delineate that oncogenic KRAS activates RAC1 which in turn leads to the production of ROS. The impaired hematopoiesis and splenomegaly that we observed when the NLRP3/IL-1β axis was active upon *KRAS* activation is very similar to the hepatosplenomegaly and pancytopenia reported in patients with activating mutations of NLRP3 that suffer from Cryopyrin-associated periodic syndrome (CAPS)^29^. In agreement with our findings, CAPS patients experience an improvement of their symptoms upon IL-1β blockade^29^. The regulation of cytokine production via oncogene/growth factor receptor activation has been previously reported for FLT3-ITD signaling blocking IL-15 production^14^, promoting lytic cell death and inflammasome activation^30^, cMYC induced IL-10^31^ and TNF-α^32^ production and EGFR induced IL-6 production^33^. The production of these cytokines may shape the tumor microenvironment in a favorable fashion for tumor cells. It is possible that besides IL-1β, also IL-18 plays a role because attenuating IL-1β with Anakinra produces a less robust protective effect compared to blocking NLRP3. Regarding the relevant cell type, the scenario that activation of NLRP3 in non-leukemic cells contributes as a driver of the disease is conceivable.

We also clarify the mechanism by showing that KRAS-induced ROS production leads to NLRP3 activation. Previous work had shown that ROS can activate NLRP3^34^. Functional inhibition of NADPH oxidase-derived ROS prevented ATP-induced caspase-1 activation and IL-1β production in alveolar macrophages^35^. Consistently we had observed NADPH gene expression being increased upon *KRAS* activation and that blocking ROS production reduced caspase-1 activation and IL-1β cleavage in KRAS-mutant mouse and human cells which has important clinical implications.

Our findings support the concept that oncogenic KRAS does not only act via its oncogenic driver function but also enhances activation of the NLRP3/IL-1β axis, which explains the clinical features of JMML and CMML patients suffering from inflammation-related symptoms. Even when NLRP3 inhibition or IL-1R blockade could not eliminate the malignant clone, this therapy could reduce the symptoms of patients such as massive splenomegaly, unexplained fever and failure to thrive, which would be a major benefit for the patients and bring them into a condition that is more stable before undergoing the potentially curative allo-HCT.

In summary, the data suggest NLRP3 and IL-1R inhibition as a strategy for the treatment of KRAS-driven hematological malignancies.

## Methods

### Human subjects

Human sample collection and analysis were approved by the Institutional Ethics Review Board of the Medical center, University of Freiburg, Germany (protocol numbers 10024/13, 26/11, 509/16). Written informed consent was obtained from each patient. All analysis of human data was carried out in compliance with relevant ethical regulations. The characteristics of patients are listed in Supplementary Tables S1–S3.

### Mice

C57BL/6 (H-2Kb, Thy-1.2) were purchased from Janvier Labs (France). *Rosa26*-Cre-ER^T2^ transgenic^36^ and *LSL-Kras*^*G12D*^ knock-in mice^37^ were maintained on a C57BL/6N background and mated to generate *Rosa26*-Cre-ER^T2^; *LSL-Kras*^*G12D*^ animals. As *Kras* deficiency is embryonic lethal and because the *LSL-Kras*^*G12D*^ *knock-in* allele prevents expression of oncogenic *Kras* prior to Cre mediated recombination, all *LSL-Kras*^*G12D*^ carrying mice retained a wildtype *Kras* allele. *Nlrp3*^−/−^ mice were obtained from the local stock at the animal facility of University Medical Center Freiburg. *Rosa26*-Cre-ER^T2^; *LSL-Kras*^*G12D*^ were bred to *Nlrp3*^−/−^ mice (both on C57BL/6 background) in the animal facility at the University of Freiburg, to generate the strain termed *Rosa26*-Cre-ER^T2^; *LSL-Kras*^*G12D*^; *Nlrp3*^−/−^. Mice were bred and housed under specific pathogen-free (SPF) conditions in the animal facilities of University Medical Center Freiburg (ZKF, Neurozentrum and IMMZ) and were used between 8 and 16 weeks of age at the time of the experiments. All mouse experiments were approved by the Federal Ministry for Nature, Environment and Consumers’ Protection of the state of Baden-Württemberg, Germany (Protocol numbers: G-17/049, G-17/093, X-14/07H, X-15/09H, X-15/10A, X-18/10C).

### Isolation of patient-derived peripheral blood mononuclear cells (PBMCs)

PBMCs were isolated from patients’ blood by Ficoll gradient centrifugation, according to the manufacturer’s protocol (Sigma-Aldrich), and frozen in Fetal Calf Serum (FCS) with 10% Dimethyl sulfoxide (DMSO). Once thawed, cells were plated in 6-well plates at 0.5 × 10^6^ cells per well, and left for 24 h to recover. If stated, PBMCs were stimulated with 200 ng/ml of LPS followed by 5 mM of ATP, with a minimum of 4 h in between. Cells were gated on CD11b^+^ cells.

### Bone marrow transplantation (BMT) and generation of BM chimera

Recipient mice were lethally irradiated with split doses of 5 Gy and 4.5 Gy (137 Cs source), with a minimum period of 4 h in between, followed by retro-orbital venous plexus injection of 5 × 10^6^ donor BM cells. Peripheral blood (PB) samples were obtained at different time points, and PB counts were performed using the scil Vet abc analyzer (Henry Schein). For BMT studies, the experiment was performed 3 times and pooled data of the indicated number of biological replicates per group is shown.

### Treatments

Tamoxifen free base (Sigma T5648) was prepared as 20 mg/ml in corn oil and administrated to mice at a single dose of 1 mg per mouse, via oral gavage. The administration of tamoxifen induces nuclear translocation of Cre-ER^T2^, causing the deletion of the LSL elements in Cre-ER^T2^, *Kras*^*G12D*^ mice^37^. A proof of successful recombination was determined using the polymerase chain reaction (PCR) protocol established by the Jacks laboratory (https://jacks-lab.mit.edu/protocols/genotyping/kras_cond) and Western blotting **(**Supplementary Fig. S1A, B). To assess the role of NLRP3 and IL-1 (IL-1 α and ß) in the disease phenotype, mice were treated with an IL-1 type 1 receptor antagonist or a NLRP3 inflammasome inhibitor. Anakinra (Kineret®; Swedish Orphan Biovitrum - sobi) was administered once daily via subcutaneous injection at a dose of 10 mg/kg. MCC950^38^ was administered once daily via intraperitoneal injection at a dose of 30 mg/kg. Mice from the vehicle group were treated with phosphate-buffered saline (PBS). Both Anakinra and NLRP3 inhibitor (MCC950) treatment started 3 weeks post-tamoxifen and ended one day before sacrificing the mice (treatment was in total for 4 weeks).

### Generation of bone marrow-derived dendritic cells (BMDCs) and stimulation

Bone marrow cells were cultured at 6 × 10^6^ cells in the presence of 40 ng/ml GM-CSF in 10 ml of RPMI 1640 medium (Gibco) in 10 cm dishes. On day 3, 10 ml of fresh medium containing 40 ng/ml GM-CSF was added. On day 7, 10 ml medium was replaced with fresh medium containing 40 ng/ml GM-CSF. Cells were used on days 7 or 8 with a CD11c^+^ purity of >80%, as determined by flow cytometry. If stated, BMDCs were stimulated with 200 ng/ml of Lipopolysaccharide (LPS, Sigma-Aldrich) for 4 h, followed by 5 mM of Adenosine 5′-triphosphate disodium salt (ATP; Sigma A7699-1G) for 30–60 min. For ELISA and colorimetric assay, BMDCs were primed with 200 ng/ml *E.coli* K12 ultra-pure LPS (InvivoGen) for 3–9 h and subsequently stimulated with either 5 µM of Nigericin (Sigma-Aldrich) for 90 min, 5 mM of ATP for 90 min or left without secondary stimulus. All stimulations were performed at least in biological and technical triplicates.

### Immunohistochemical and histological analysis

Tissue sections of tibia, femur, and spleen were collected at the terminal point of the experiment, fixed in 4% paraformaldehyde for 12 to 16 h at 4 °C and washed in 70% ethanol. Tissues were embedded in paraffin and cut into 5-μm sections, followed by the standard protocol of Hematoxylin/Eosin (H/E) staining. Blood smears were stained with Pappenheim stain. The results were assessed, graded and quantified by an investigator blinded to the experimental groups.

### Flow cytometry

The fluorochrome-conjugated antibodies mAbs (Clone) used for flow cytometric analysis are shown in Supplementary Table S4. To examine cell viability and exclude dead cells, the LIVE/DEAD Fixable Dead Cell Stain kit (Molecular Probes) or the Zombie NIR Fixable Viability kit (Biolegend) were used. For intracellular staining, the BD Cytofix/Cytoperm kit (BD Biosciences) was used following the manufacturer’s protocol. Data were acquired on a BD LSR Fortessa flow cytometer (BD Bioscience) and analyzed using the FlowJo (Flowjo 10.4, LLC) software. Multiparameter high-dimensional data was acquired on a FACSSymphony (BD Bioscience) and compensated in FlowJo (V10.6), then live, single CD45^+^ cells were exported and analyzed using the R environment^39^. Data were processed for FlowSOM clustering^40^. For dimensionality reduction the UMAP package was used^41^.

### Western blot

Cells were lysed in radioimmunoprecipitation assay buffer (Santa Cruz Biotechnology) supplemented with Phosphatase Inhibitor Cocktail 2 (Sigma-Aldrich) and protein concentrations were determined using the Pierce BCA Protein Assay Kit (Life Technologies). Cell lysates prepared for SDS-PAGE using NuPAGE™ LDS sample buffer and NuPAGE™ sample reducing agent (Invitrogen). Supernatant samples from cell-free supernatants were prepared using sample buffer containing SDS and Dithiothreitol (DTT). The primary antibodies were used against caspase-1 (p20) (mouse; Adipogen #AG-20B-0042-C100), caspase-1 (p20) (human; Adipogen #AG-20B-0048-C100), cleaved-IL-1β (Asp117) (Cell Signaling Technology #52718), IL-1β (R&D Systems #AF-401), Ras (Abcam #52939), active Rac1 (NewEast Biosciences #26903). β-actin (Cell Signaling Technology #4970, or Santa Cruz Biotechnology #sc-47778) or Vinculin (Cell Signaling Technology #13901) were used as loading controls. As a secondary antibody, horseradish peroxidase (HRP)-linked anti-rabbit IgG or anti-mouse IgG were used (#7074 and #7076S Cell Signaling Technology). The blot signals were detected using WesternBright Quantum HRP substrate (Advansta), imaged using ChemoCam Imager 3.2.0 (Intas Science Imaging Instruments GmbH) and quantified using ImageJ (NIH) software.

### Reactive oxygen species (ROS) analysis and inhibition

2′, 7′-dichlorofluorescein diacetate (H2DCF-DA; Sigma D6883) was used to detect cellular ROS. One vial was dissolved in dimethyl sulfoxide (DMSO) to prepare 20 mg/ml stock solution. Briefly, cells were pelleted and resuspended in 1.16 µg/ml of H2DCF-DA and incubated at 37 °C in the dark for 30 min. The cells were pelleted and resuspended in fresh medium along with stimulus and incubated at 37 °C in the dark for 60 min. After washing, cells were stained to their corresponding surface antigen at 4 °C in the dark for 20 min and washed before acquisition on flow cytometer. MitoSOX^TM^ red reagent (Thermofisher M36008) was used as an indicator of mitochondrial ROS (mtROS) in live cells. One vial was dissolved in DMSO to make a 5 mM MitoSOX^TM^ stock solution. Cells were incubated in 5 µM MitoSOX^TM^ in HBSS/Ca^2+^/Mg^2+^ solution (Gibco 14025) at 37 °C for 30 min. ROS signal was measured with a BD LSR Fortessa flow cytometer and analyzed using FlowJo software. For inhibition of ROS, 30 μM of Ebselen (Enzo Life Sciences), 50 μM ammonium pyrrolidinedithiocarbamate (PDTC; Enzo Life Sciences) or DMSO as a control, were added 2.5–3 h after priming with LPS and 20–30 min before stimulation with ATP.

### Microarray analysis

RNA was isolated from BMDCs using the RNeasy Mini Kit (Qiagen). RNA quality was assessed by Agilent 2100 Bioanalyzer (Agilent 141 Technologies). RNA samples with an RNA integrity number (RIN) greater than 8 were further processed. Affymetrix Clariom S Mouse arrays were normalized using Single-Channel Array Normalization and exon expression summarized to the gene level using the R/Bioconductor package pd.clariom.s.mouse. Differential gene expression analysis between *Kras*^*G12D*^ BM versus WT was calculated using the R/Bioconductor limma package with p-values corrected for multiple testing using Benjamini & Hochberg. Gene set enrichment analysis (GSEA) was performed using the R/Bioconductor package GAGE which tests for significant differential regulation of a gene set instead of individual genes. The complete gene expression data are available in the GEO repository under the access ID GSE131885 (https://www.ncbi.nlm.nih.gov/geo/query/acc.cgi?&acc=GSE131885).

### Ras downstream effector pathways inhibition

To delineate the role of Ras effector pathways leading to NLRP3 activation, MEK-specific inhibitors (Trametinib (GSK1120212) and Selumetinib (AZD6244); Selleckhem S2673 and S1008), an ERK inhibitor (Ulixertinib (BVD-523); Selleckhem S7854), PI3K-specific inhibitors (Buparlisib (BKM120) and Pictilisib (GDC-0941); Selleckhem S2247 and S1065) and a Rac1 inhibitor (NSC 23766; Tocris Bioscience 2161) were used. DMSO was used as a control. In brief, BMDCs generated from *Kras*^*G12D*^ BM mice were treated with the inhibitors or DMSO as a control with the indicated concentrations and times, and later analyzed by flow cytometry or lysed for western blot.

### Proliferation analysis

On day 7, the number of BMDCs generated from WT or *Kras*^*G12D*^ mice was counted per 10 cm dish. Biological replicates each mouse was considered, and the average was taken. For the proliferation analysis, the Click-iT™ EdU Pacific Blue™ Flow Cytometry Assay kit (Invitrogen™; C10418) was used, following the manufacturer’s protocol. Briefly, 10 µM of EdU was added to the culture medium and incubated for 1–2 h. After cells were harvested and washed with 1% BSA in PBS, extracellular staining proceeded. Following, cells were fixed using the Click-iT™ fixative for 15 min at room temperature, and subsequent washes were performed using the 1X Click-iT™ saponin-based permeabilization and wash reagent. Lastly, cells were incubated for 30 min at room temperature protected from light with the Click-iT™ reaction cocktail composed of CuSO_4_, Pacific Blue dye, reaction buffer additive and PBS, following the manufacturer’s instructions. Cells were later analyzed by flow cytometry.

### Cell isolation and DNA extraction

Using fluorescence-activated cell sorting, the CD11b^+^ cell population was sorted from PBMCs previously isolated from Kras^mut^ patients (BD FACSAria™ III, BD Biosciences). Cells were lysed using RLT plus lysis buffer (Qiagen) and DNA extraction was performed on a QIAsymphony robot (Qiagen).

### Targeted Next Generation Sequencing (NGS)

The Illumina TruSight Tumor 15 and TruSight Myeloid Panels were used for targeted resequencing and processed as described by the manufacturer (Illumina Inc.). Sequencing libraries were sequenced paired-end on an Illumina NextSeq 550 with 2 × 150 base reads and FASTQ-files were further analyzed with the SeqNext software (JSI Medical Systems). We used a significance threshold of 3% for the detection of missense mutations, with a minimum coverage of 500 reads and 50 reads per variant.

### Digital droplet polymerase chain reaction (ddPCR)

ddPCR was used to validate selected mutations in KRAS (G12V, G12A). The ddPCR reaction volume was 20 µL and composed of 10 µL of dPCR supermix for probes (Bio-Rad Laboratories GmbH), 900 nmol/L of each primer and 250 nmol/L probes. Each reaction mixture was partitioned into approximately 20,000 droplets using a QX200 automated droplet generator (Bio-Rad Laboratories GmbH) and then cycled under set conditions. Cycled droplets were read in the QX 200 droplet-reader and the analysis of the data was performed using the QuantaSoft analysis software (Version 1.0, Bio-Rad Laboratories GmbH). The threshold between the positive and negative droplet clusters was manually set for both fluorochrome channels for each sample. Data analysis was performed in those samples were 10,000 or more accepted droplets were obtained.

### Lactate dehydrogenase (LDH) release assay and enzyme-linked immunosorbent assay (ELISA)

Lytic cell death was quantified by determining LDH levels in the cell-free supernatant using the CytoTox 96 Non-Radioactive Cytotoxicity kit (Promega G1780), according to the manufacturer’s instructions. Absorbance of medium was measured to serve as blank value and was subtracted from the sample values. Results are shown as percentage of 100 % dead cells after lysis with 0.8% Triton X-100 for 45 min. IL-1β quantification of cell-free supernatants was performed using ELISA, following the manufacturer’s instructions (Invitrogen 88701377). Measurements were performed at least in technical triplicates and absorbance was detected using a microplate reader.

### Reticulocyte counting

Reticulocytes were distinguished from red blood cells by 2 criteria:

1. Size: reticulocytes are larger than erythrocytes: 10–15 mm vs. 6–8 mm
2. Morphology: Reticulocytes have a scattered reticulum network in the cytoplasm which is visible as a blue granular precipitate.

All experiments were reproducible and were performed at least 3 times.

### Statistical analysis

GraphPad Prism v7.01 was used for statistical analysis. A sample size of at least n = 5 per group was determined by 80% power to reach a statistical significance of 0.05 to detect an effect size of at least 1.06. For statistical analysis an unpaired *t*-test (two-sided) was applied. All data were tested for normality applying the Kolmogorov–Smirnov test. If the data did not meet the criteria of normality, the Mann–Whitney *U* test was applied. Data are presented as mean and s.e.m. (error bars). Differences were considered significant when the *p*-value was <0.05.

### Reporting summary

Further information on research design is available in the Nature Research Reporting Summary linked to this article.

## Supplementary information

Supplementary Figures and tables

Reporting Summary

## Data Availability

Microarray data are deposited in the database GEO repository under the access ID GSE131885. The source data underlying Figs. 1C–F, 1H, 2B, C, 2F, 2H, 3B, 3D, 3F, G, 4B, C, 4F, G, 5A–C, 5F, 5H, I, 6A–F, 6H, 6J, 7C–K, 8A, B, 8D, E, 8G, I, 9B, 9D, 9E–G, 9I–L and Supplementary Figs. S1A–C, S1G, S2B–C, S4B–E, S5F–I are provided as a Source Data file.

## Source data

Source data
