# Supplementary material for: Oncogenic KrasG12D causes myeloproliferation via NLRP3 inflammasome activation
Source: Nat Commun. 2020 Apr 3;11:1659. doi: 10.1038/s41467-020-15497-1 (PMC7125138; doi:10.1038/s41467-020-15497-1)
Supplement: Supplementary file 1 — Supplementary Figures and tables [file 41467_2020_15497_MOESM1_ESM.pdf]

Oncogenic Kras^{G12D} causes myeloproliferation via NLRP3 inflammasome activation

Hamarsheh et al.

Fig. S1

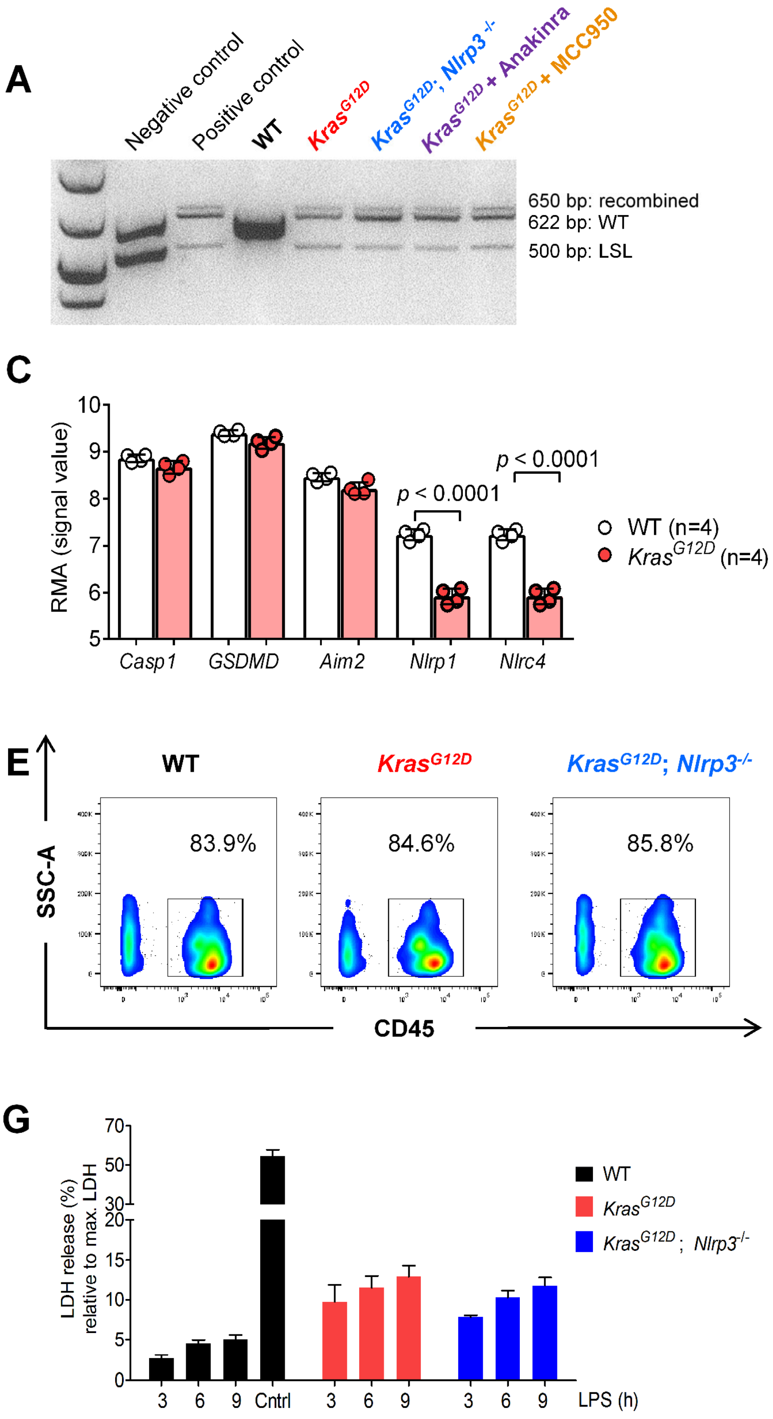

Supplementary Fig. S1 Proof of successful Cre- recombination and engraftment

(A) Proof of successful Cre- recombination in C57BL/6 recipient mice that have undergone transplantation of WT, *Kras*^{G12D}, *Kras*^{G12D} or *Nlrp3*$^{-/-}$, *Kras*^{G12D} BM and that were treated with tamoxifen. The image is representative for three independent experiments.

(B) Proof of the increased Ras expression in BMDCs derived from *Kras*^{G12D} mice compared to WT littermates, detected by western blot. The image is representative for four independent experiments.

(C) Expression of *Casp1*, *GSDMD*, *Aim2*, *Nlrp1* and *Nlrc4* in BMDCs isolated from WT (n=4) or *Kras*^{G12D} (n=4) mice following treatment by tamoxifen, as analyzed by microarray. Data are shown as mean ± SEM.

(D) Representative flow cytometry plot showing the gating strategy used for CD11c$^+$ BMDCs generated from WT, *Kras*^{G12D} or *Kras*^{G12D}; *Nlrp3*$^{-/-}$ BM mice.

(E) Representative flow cytometry plots showing the frequency of CD45$^+$ cells in WT, *Kras*^{G12D} or *Kras*^{G12D}; *Nlrp3*$^{-/-}$ BM mice, as quantified 4 weeks post-transplantation and prior to tamoxifen treatment.

(F) Representative flow cytometry plot showing the engraftment efficiency in chimera mice following transplantation of WT, *Kras*^{G12D} or *Kras*^{G12D} and *Nlrp3*$^{-/-}$ BM (CD45.2) into letally irradiated WT recipient mice (CD45.1).

(G) The graph displays the levels of LDH release in BMDCs generated from WT, *Kras*^{G12D} and *Kras*^{G12D}; *Nlrp3*$^{-/-}$ BM mice, quantified from cell-free supernatants by colorimetric assay, following LPS-priming for the indiciated times. The control sample (Cntrl) was LPS-primed for 4.5 hours in addition to Nigericin. Data points are shown as mean ± SD, and are representative of 3 biological replicates.

Fig. S2

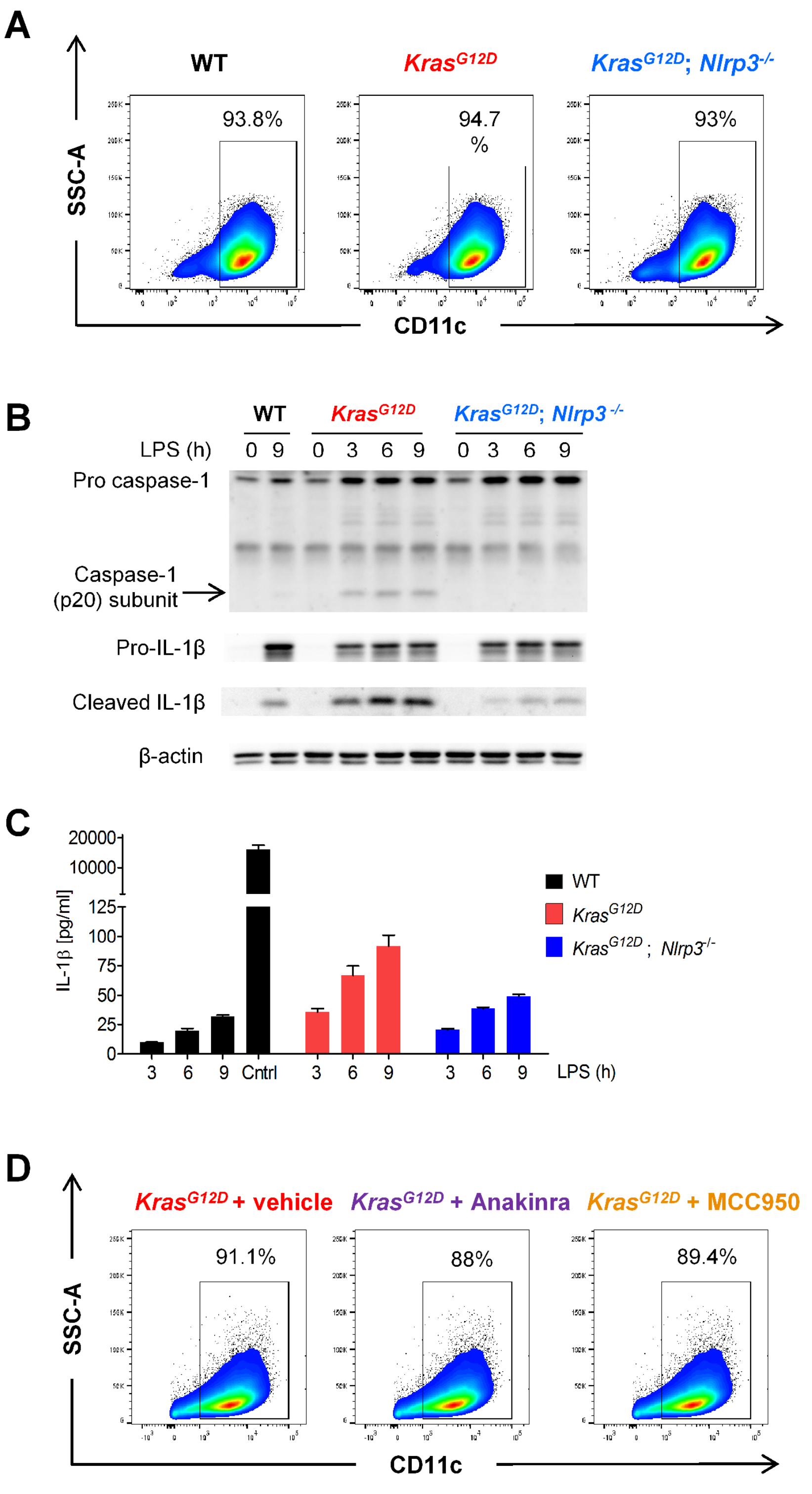

Supplementary Fig. S2 CD11c+ BMDCs, after isolation of BM from WT, *Kras*^{G12D} or *Kras*^{G12D}; *Nlrp3*$^{-/-}$ BM mice

(A) Representative flow cytometry plots showing the normal generation of $CD11c^+$ BMDCs, after isolation of BM from WT, *Kras*^{G12D} or *Kras*^{G12D}; *Nlrp3*$^{-/-}$ recipient mice.
(B) Western blot shows the amount of caspase-1 (p20 subunit) and cleaved IL-1β analysed in cell-free supernatants, and amount of pro-IL-1β analysed in cell lysates of BMDCs generated from WT, *Kras*^{G12D} or *Kras*^{G12D}; *Nlrp3*$^{-/-}$ BM mice, following LPS-priming for the indiciated times. The blot is representative for three independent experiments.
(C) The graph displays the levels of IL-1β in BMDCs generated from WT, *Kras*^{G12D} or *Kras*^{G12D}; *Nlrp3*$^{-/-}$ BM mice, quantified from cell-free supernatants by ELISA, following LPS-priming for the indiciated times. The control sample (Cntrl) was LPS-primed for 4.5 hours in addition to Nigericin. Data points are shown as mean ± SD, and are representative of 3 biological replicates.
(D) Representative flow cytometry plots showing the normal generation of $CD11c^+$ BMDCs, after isolation of BM from *Kras*^{G12D} BM mice treated with vehicle, Anakinra or MCC950.

Fig. S3

A

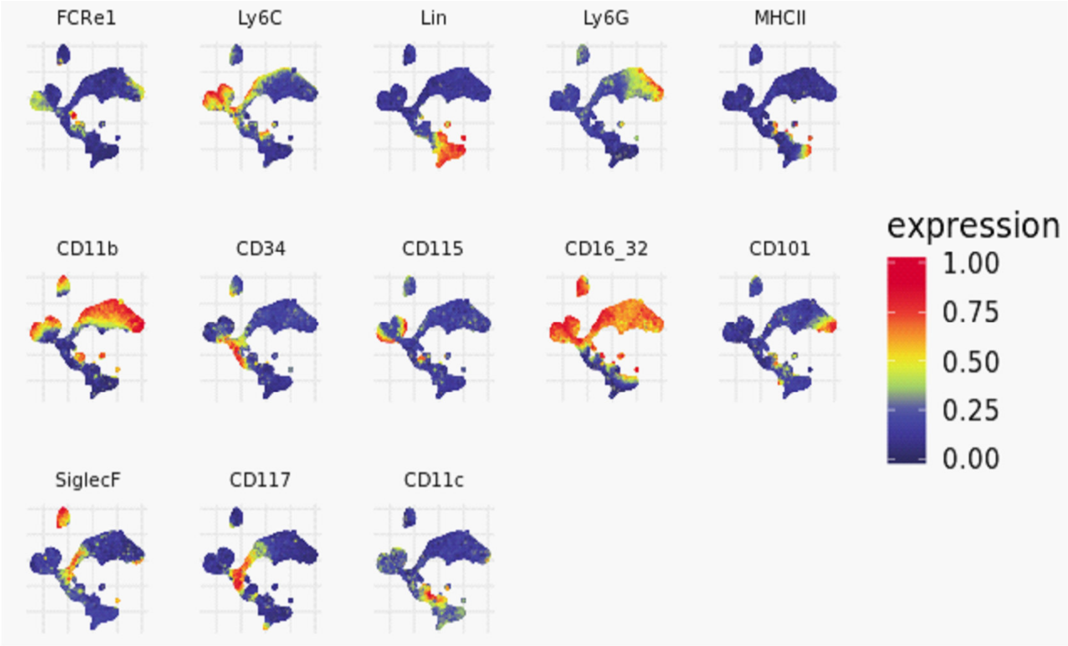

B

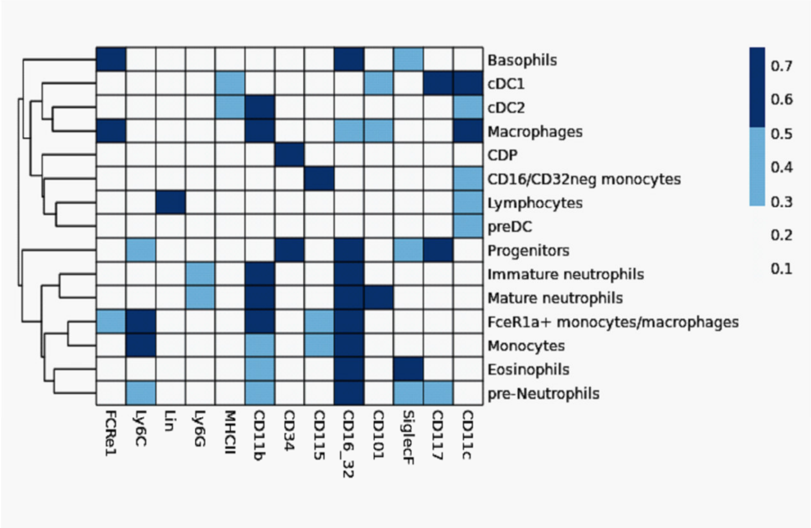

Supplementary Fig. S3 UMAP plot and heat map for each annotated population after FlowSOM-guided metaclustering.

(A) UMAP plot displaying expression of the indicated markers on stochastically selected BM $CD45^+$ leukocytes (gated on live/single cells/CD45) from vehicle-treated $Kras^{G12D}$ (n=4), Anakinra-treated $Kras^{G12D}$ (n=5) or MCC950-treated $Kras^{G12D}$ (n=5) BM mice. Lin = lymphocyte lineage cocktail containing anti-CD19, anti-CD3, anti CD49b and anti-TER119.

(B) The heat map shows the median marker expression (value range: 0-1) for each annotated population after FlowSOM-guided metaclustering.

Fig. S4

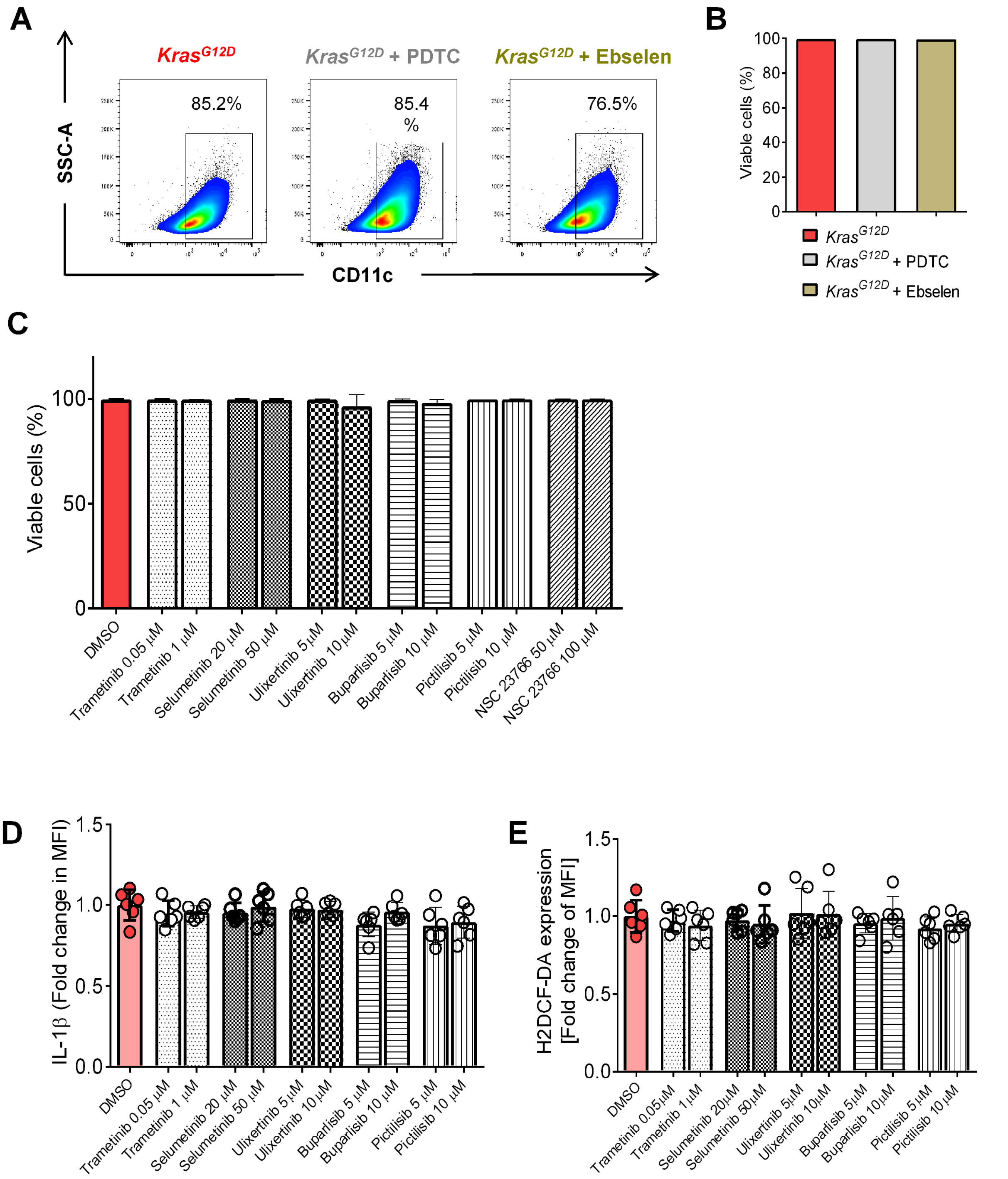

Supplementary Fig. S4 Treatment of *Kras^{G12D}* BMDCs with ROS inhibitors or MEK, ERK and PI3K inhibitors

(A) Representative flow cytometry plots showing the normal generation of CD11c$^+$ BMDCs, after isolation of BM from *Kras*^{G12D} BM mice and treatment with DMSO or ROS inhibitors PDTC and Ebselen**.**

(B) The graph shows the percentage of viable cells in CD11c$^+$ BMDCs generated from *Kras*^{G12D} BM mice, after treatment with ROS inhibitors PDTC and Ebselen.

(C) The graph shows the percentage of viable cells in CD11c$^+$ BMDCs generated from *Kras*^{G12D} BM mice, after treatment with DMSO, 0.05 μM or 1 μM of MEK-specific inhibitor Trametinib, 20 or 50 μM of MEK-specific inhibitor Selumetinib, 5 or 10 μM of ERK-specific inhibitor Ulixertinib, 5 or 10 μM of PI3K-specific inhibitor Buparlisib. 5 or 10 μM of PI3K-specific inhibitor Pictilisib for 30 hours, 50 or 100 μM of RAC1 inhibitor NSC 23766 for 1 hour.

(D) The graph shows the fold change of IL-1β expression as measured by flow cytometry in CD11c$^+$ BMDCs generated from *Kras*^{G12D} BM mice, after treatment with DMSO, 0.05 μM or 1 μM of MEK-specific inhibitor Trametinib, 20 or 50 μM of MEK-specific inhibitor Selumetinib, 5 or 10 μM of ERK-specific inhibitor Ulixertinib, 5 or 10 μM of PI3K-specific inhibitor Buparlisib, 5 or 10 μM of PI3K-specific inhibitor Pictilisib for 30 hours, and stimulation with 200 ng/ml LPS and 5 mM ATP, normalized to DMSO-treated *Kras*^{G12D} BMDCs.

(E) The graph shows the fold change of H2DCF-DA expression as measured by flow cytometry in CD11c$^+$ BMDCs generated from *Kras*^{G12D} BM mice, after treatment with DMSO, 0.05 μM or 1 μM of MEK-specific inhibitor Trametinib, 20 or 50 μM of MEK-specific inhibitor Selumetinib, 5 or 10 μM of ERK-specific inhibitor Ulixertinib, 5 or 10 μM of PI3K-specific inhibitor Buparlisib, 5 or 10 μM of PI3K-specific inhibitor Pictilisib for 30 hours, and stimulation with 200 ng/ml LPS and 5 mM ATP, normalized to DMSO-treated *Kras*^{G12D} BMDCs.

All data are shown as mean ± SEM.

Fig. S5

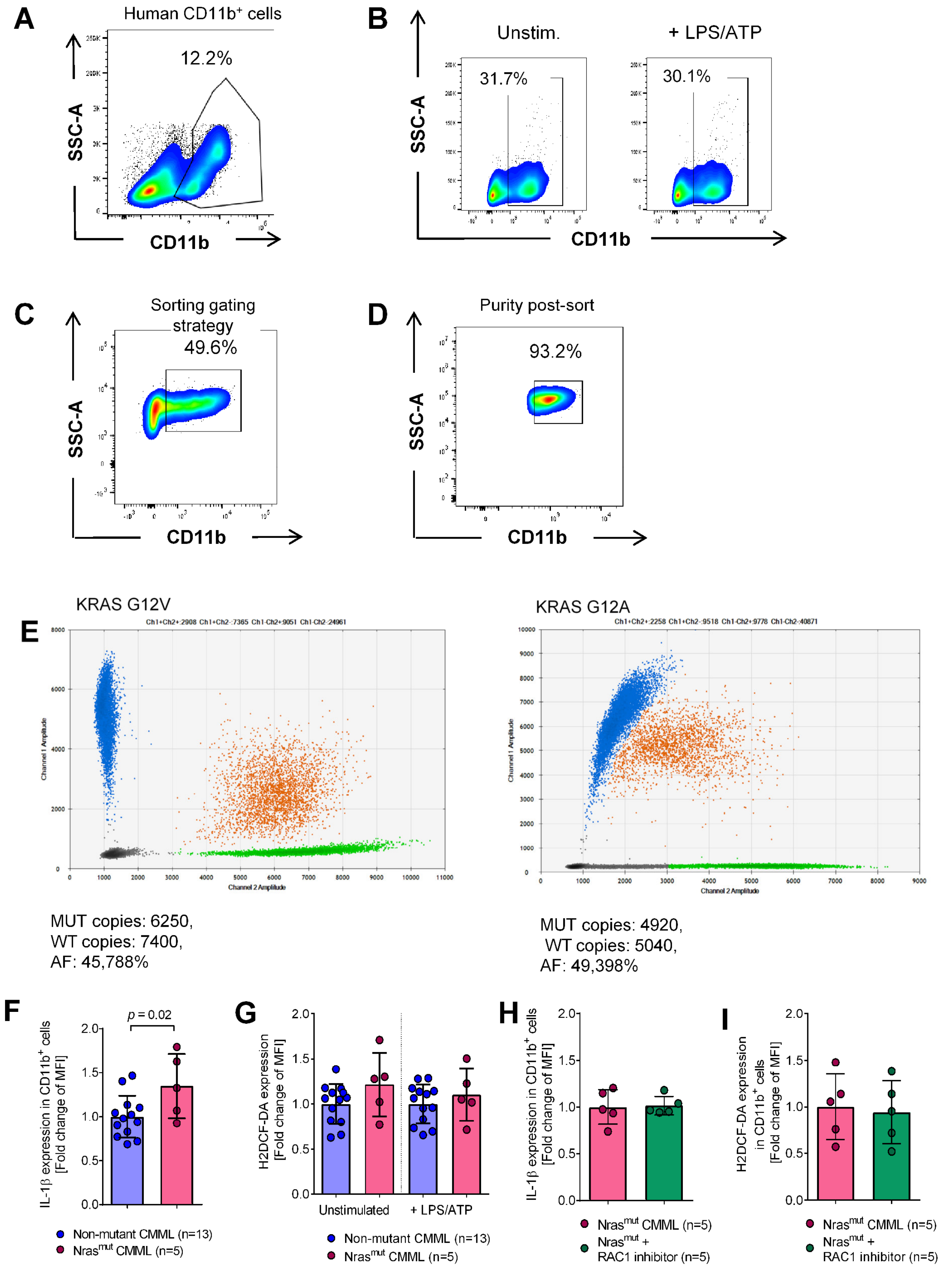

A
Human CD11b+ cells
12.2%
SSC-A
CD11b
B
Unstim.
+ LPS/ATP
31.7%
30.1%
SSC-A
CD11b
C
Sorting gating strategy
49.6%
SSC-A
CD11b
D
Purity post-sort
93.2%
SSC-A
CD11b
E
KRAS G12V
Channel 1 Amplitude
Channel 2 Amplitude
MUT copies: 6250,
WT copies: 7400,
AF: 45,788%
KRAS G12A
Channel 1 Amplitude
Channel 2 Amplitude
MUT copies: 4920,
WT copies: 5040,
AF: 49,398%
F
p = 0.02
IL-1β expression in CD11b+ cells [Fold change of MFI]
Non-mutant CMML (n=13)
Nras mut CMML (n=5)
G
H2DCF-DA expression [Fold change of MFI]
Unstimulated
+ LPS/ATP
Non-mutant CMML (n=13)
Nras mut CMML (n=5)
H
IL-1β expression in CD11b+ cells [Fold change of MFI]
Nras mut CMML (n=5)
Nras mut + RAC1 inhibitor (n=5)
I
H2DCF-DA expression in CD11b+ cells [Fold change of MFI]
Nras mut CMML (n=5)
Nras mut + RAC1 inhibitor (n=5)

Supplementary Fig. S5 Analysis of PBMCs isolated from Kras^{mut}, Nras^{mut} or non-mutant patients

(A) Representative flow cytometry plot showing the gating strategy used for CD11b^{+} PBMCs isolated from Kras^{mut} or non-Kras^{mut} patients.

(B) Representative flow cytometry plots showing the gating strategy used for CD11b^{+} PBMCs, and confirming no effect on the cells following stimulation with LPS and ATP.

(C) Representative flow cytometry plot showing the gating strategy and purity of sorted CD11b^{+} cells from Kras^{mut} patients PBMCs.

(D) Representative flow cytometry plot confirming the purity of sorted CD11b^{+} cells from Kras^{mut} patients PBMCs.

(E) Representative ddPCR results showing a two-dimensional (2D) scatter plot with the four clusters obtained with mutant and wild-type alleles. The number of events is very high which represents a very high copy number of mutant *Kras G12V or G12A* molecules, respectively in the specimen (blue: droplets contain only mutant template; orange: droplets contain both templates; black: droplets contain no template, green: droplets contain only wild-type template).The plot is representative of 3 independent experiments.

(F) The graph displays the fold change of IL-1β expression as measured by flow cytometry in CD11b^{+} cells of CMML patients with an *NRAS* mutation (Nras^{mut}) (n=5), or without an *NRAS* mutation (non-mutant) (n=13), after stimulation with 200 ng/ml LPS and 5 mM ATP, normalized to non-mutant.

(G) The graph displays the fold change of H2DCF-DA expression as measured by flow cytometry in CD11b^{+} cells of Nras^{mut} (n=5) and non-mutant (n=13) CMML patients, normalized to non-mutant in both stimulation conditions.

(H) The graph displays the fold change of IL-1β expression as measured by flow cytometry in CD11b^{+} cells of CMML patients with an *NRAS* mutation (Nras^{mut}) treated with DMSO (n=5) or 100 µM of RAC1 inhibitor NSC 23766 (n=5), after stimulation with 200 ng/ml LPS and 5 mM ATP, normalized to DMSO-treated Nras^{mut}.

(I) The graph displays the fold change of H2DCF-DA expression as measured by flow cytometry for in CD11b^{+} cells of CMML patients with an *NRAS* mutation (Nras^{mut}) treated with DMSO (n=5) or 100 µM of RAC1 inhibitor NSC 23766 (n=5), after stimulation with 200 ng/ml LPS and 5 mM ATP, normalized to DMSO-treated Nras^{mut}.

Supplementary Tables

Suppl. Table S1: Characteristics of JMML patients

**Total number of patients**	9
**Variable**	**Median (Range)**
**Age**	4 (0.5-7.6)
	**% (absolute number)**
**Gender**	
Female	33.3 (3)
Male	66.7 (6)
**Diagnosis**	
JMML	100 (9)
***KRAS* mutation**	
Yes	100 (9)

Abbreviations: JMML = Juvenile Myelomonocytic Leukemia

Suppl. Table S2: Characteristics of AML patients

**Total number of patients**	33
**Variable**	
	**Median (Range)**
**Age**	61 (25-80)
	**% (absolute number)**
**Gender**	
Female	51.5 (17)
Male	48.5 (16)
**Diagnosis**	
AML	100 (33)
***KRAS* mutation**	
Yes	33.3 (11)
No	66.7 (22)

Abbreviations: AML = Acute Myeloid Leukemia

Suppl. Table S3: Characteristics of CMML patients

**Total number of patients**	23
**Variable**	**Median (Range)**
**Age**	75 (55-89)
	**% (absolute number)**
**Gender**	
Female	39.1 (9)
Male	60.9 (14)
**Diagnosis**	
CMML	100 (23)
***RAS* mutation**	
KRAS	21.7 (5)
NRAS	21.7 (5)
No *RAS* mutation	56.5 (13)

Abbreviations: CMML = Chronic myelomonocytic leukemia

Suppl. Table S4: Antibodies for flow cytometry

Antibody	Clone	Catalogue number	Flourochrome	Vendor
Anti-mouse CD11b	M1/70	557397	PE	BD Bioscience
Anti-mouse CD11b	M1/70	564443	BUV 737	BD Bioscience
Anti-mouse/human CD11b	M1/70	101205	FITC	Biolegend
Anti-mouse/human CD11b	M1/70	101224	PB	Biolegend
Anti-mouse CD11c	N418	117306	FITC	Biolegend
Anti-mouse CD11c	N418	117322	PB	Biolegend
Anti-mouse CD11c	N418	35-0114-82	PE-Cy5.5	eBioscience
Anti-mouse CD45	30-F11	103108	FITC	Biolegend
Anti-mouse CD45	30-F11	103126	PB	Biolegend
Anti-mouse CD45	30-F11	565967	BUV 395	BD Bioscience
Anti-mouse CD45.1	A20	110706	FITC	Biolegend
Anti-mouse CD45.2	104	109820	PB	Biolegend
Anti-mouse CD45R/B220	RA3-6B2	103247	BV510	Biolegend
Anti-mouse CD45R/B220	RA3-6B2	103212	APC	Biolegend
Anti-mouse CD90.2	30-H12	105326	PE-Cy7	Biolegend
Anti-mouse CD90.2	30-H12	105307	PE	Biolegend
Anti-mouse CD34	SA376A4	152208	BV 421	Biolegend
Anti-mouse CD115	AFS98	135517	BV 605	Biolegend
Anti-mouse CD101	Moushi101	12-1011-82	PE	eBioscience
Anti-mouse CD117	2B8	105810	PE-Cy5	Biolegend
Anti-mouse CD19	eBio1D3	17-0193-82	APC	eBioscience
Anti-mouse CD3	17A2	100236	APC	Biolegend
Anti-mouse CD49b	DX5	108910	APC	Biolegend
Anti-mouse CD16/32	93	101337	BV 711	Biolegend
Anti-mouse Ly-6C	AL-21	560525	PerCP Cy5.5	BD Bioscience
Anti-mouse FceRIa	MAR-1	134329	AlexaFlour 488	Biolegend
Anti-mouse Ly6G	1A8	565707	BUV 563	BD Bioscience
Anti-mouse I-A/I-E (MHC class II)	M5/114.15.2	750280	BUV 661	BD Bioscience
Anti-mouse TER119	Ter-119	17-5921-81	APC	eBioscience
Anti-mouse Siglec-F	E50-2440	562757	PE-CF594	BD Bioscience
Anti-mouse IL-1β Pro-form	NJTEN3	12-7114-80	PE	eBioscience
Anti-human IL-1β	CRM56	12-7018-41	PE	eBioscience

Abbreviations:

PB: Pacific blue
FITC: Fluorescein isothiocyanate
PE: Phycoerythrin
APC: Allophycocyanin
-Cy: -Cyanine
BV-: Brilliant Violet
PerCP-: Peridinin-chlorophyll-protein
BUV-: Brilliant Ultraviolet
