# Supplementary material for: Oncogenic KrasG12D causes myeloproliferation via NLRP3 inflammasome activation
Source: Nat Commun. 2020 Apr 3;11:1659. doi: 10.1038/s41467-020-15497-1 (PMC7125138; doi:10.1038/s41467-020-15497-1)
Supplement: Supplementary file 2 — Reporting Summary [file 41467_2020_15497_MOESM2_ESM.pdf]

nature research

Corresponding author(s): Robert Zeiser

Last updated by author(s): Jan 19, 2020

Reporting Summary

Nature Research wishes to improve the reproducibility of the work that we publish. This form provides structure for consistency and transparency in reporting. For further information on Nature Research policies, see Authors & Referees and the Editorial Policy Checklist.

Statistics

For all statistical analyses, confirm that the following items are present in the figure legend, table legend, main text, or Methods section.

n/a	Confirmed	
☐	☒	The exact sample size (n) for each experimental group/condition, given as a discrete number and unit of measurement
☐	☒	A statement on whether measurements were taken from distinct samples or whether the same sample was measured repeatedly
☐	☒	The statistical test(s) used AND whether they are one- or two-sided<br>*Only common tests should be described solely by name; describe more complex techniques in the Methods section.*
☒	☐	A description of all covariates tested
☐	☒	A description of any assumptions or corrections, such as tests of normality and adjustment for multiple comparisons
☐	☒	A full description of the statistical parameters including central tendency (e.g. means) or other basic estimates (e.g. regression coefficient) AND variation (e.g. standard deviation) or associated estimates of uncertainty (e.g. confidence intervals)
☐	☒	For null hypothesis testing, the test statistic (e.g. F, t, r) with confidence intervals, effect sizes, degrees of freedom and P value noted<br>*Give P values as exact values whenever suitable.*
☒	☐	For Bayesian analysis, information on the choice of priors and Markov chain Monte Carlo settings
☒	☐	For hierarchical and complex designs, identification of the appropriate level for tests and full reporting of outcomes
☒	☐	Estimates of effect sizes (e.g. Cohen's d, Pearson's r), indicating how they were calculated

Our web collection on statistics for biologists contains articles on many of the points above.

Software and code

Policy information about availability of computer code

Data collection	Not applicable.
Data analysis	All statistical analysis was performed using Graphpad Prism version 7.01. Flow cytometry data was analyzed using FlowJo software (Flowjo v10.4 or v10.6, LLC). V10.6). For Multiparameter high dimensional data. single CD45+ cells were exported and analyzed using the R environment 40. For dimensionality reduction the UMAP 42 package was used. The signals from western blots were quantified using ImageJ (NIH) software. For targeted Next Generation Sequencing (NGS), FASTQ-files were further analysed with the SeqNext software (JSI Medical Systems). Analysis of the digital droplet PCR (ddPCR) data was performed using the QuantaSoft analysis software (Version 1.0, Bio-Rad Laboratories GmbH).

For manuscripts utilizing custom algorithms or software that are central to the research but not yet described in published literature, software must be made available to editors/reviewers. We strongly encourage code deposition in a community repository (e.g. GitHub). See the Nature Research guidelines for submitting code & software for further information.

Data

Policy information about availability of data

All manuscripts must include a data availability statement. This statement should provide the following information, where applicable:

- Accession codes, unique identifiers, or web links for publicly available datasets
- A list of figures that have associated raw data
- A description of any restrictions on data availability

Microarray data are deposited in the database GEO repository under the access ID GSE131885.

October 2018

Field-specific reporting

Please select the one below that is the best fit for your research. If you are not sure, read the appropriate sections before making your selection.

☒ Life sciences ☐ Behavioural & social sciences ☐ Ecological, evolutionary & environmental sciences

For a reference copy of the document with all sections, see nature.com/documents/nr-reporting-summary-flat.pdf

Life sciences study design

All studies must disclose on these points even when the disclosure is negative.

Sample size	For the sample size throughout vivo experiments a sample size of at least n=5 per group was used.
Data exclusions	No data were excluded from the analyses.
Replication	All the experimental findings were reliably reproduced.
Randomization	There was no randomization of mice or samples before analysis. Therefore, all samples or mice were included in our analysis.
Blinding	The experiments were performed in a non-blinded fashion except for the histological scoring. In order to obtain unbiased data, the histopathological scoring and analysis was performed by a pathologist blinded to the genotype or treatment groups. Only after finalization of the quantitative scores, the samples were allocated to their designated groups.

Reporting for specific materials, systems and methods

We require information from authors about some types of materials, experimental systems and methods used in many studies. Here, indicate whether each material, system or method listed is relevant to your study. If you are not sure if a list item applies to your research, read the appropriate section before selecting a response.

Materials & experimental systems

n/a	Involved in the study	
☐	☒	Antibodies
☒	☐	Eukaryotic cell lines
☒	☐	Palaeontology
☐	☒	Animals and other organisms
☐	☒	Human research participants
☒	☐	Clinical data

Methods

n/a	Involved in the study	
☒	☐	ChIP-seq
☐	☒	Flow cytometry
☒	☐	MRI-based neuroimaging

Antibodies

Antibodies used	The fluorochrome-conjugated antibodies mAbs (Clone) used for flow cytometric analysis are: Anti-mouse CD11b (Clone: M1/70, Flourochrome: PE, Catalouge #557397, Vendor: BD Bioscience). Anti-mouse CD11b (Clone: M1/70, Flourochrome: BUV 737, Catalouge #564443, Vendor: BD Bioscience). Anti-mouse/human CD11b (Clone: M1/70, Flourochrome: FITC, Catalouge #101205, Vendor: Biolegend). Anti-mouse/human CD11b (Clone: M1/70, Flourochrome: PB, Catalouge #101224, Vendor: Biolegend). Anti-mouse CD11c (Clone: 30-F11, Flourochrome: FITC, Catalouge #117306, Vendor: Biolegend). Anti-mouse CD11c (Clone: N418, Flourochrome: PB, Catalouge #117322, Vendor: Biolegend). Anti-mouse CD11c (Clone: N418, Flourochrome: PE-Cy5.5, Catalouge #35-0114-82, Vendor: eBioscience). Anti-mouse CD45 (Clone: N418, Flourochrome: FITC, Catalouge #103108, Vendor: Biolegend). Anti-mouse CD45 (Clone: N418, Flourochrome: PB, Catalouge #103126, Vendor: Biolegend). Anti-mouse CD45 (Clone: N418, Flourochrome: BUV 395, Catalouge #565967, Vendor: BD Bioscience). Anti-mouse CD45.1 (Clone: A20, Flourochrome: FITC, Catalouge #110706, Vendor: Biolegend). Anti-mouse CD45.2 (Clone: 104, Flourochrome: PB, Catalouge #109820, Vendor: Biolegend). Anti-mouse CD45R/B220 (Clone: RA3-6B2, Flourochrome: BV510, Catalouge #103247, Vendor: Biolegend). Anti-mouse CD45R/B220 (Clone: RA3-6B2, Flourochrome: APC, Catalouge #103212, Vendor: Biolegend). Anti-mouse CD90.2 (Clone: 30-H12, Flourochrome: PE-Cy7, Catalouge #105326, Vendor: Biolegend). Anti-mouse CD90.2 (Clone: 30-H12, Flourochrome: PE, Catalouge #105307, Vendor: Biolegend). Anti-mouse CD34 (Clone: SA376A4, Flourochrome: BV 421, Catalouge #152208, Vendor: Biolegend). Anti-mouse CD115 (Clone: AFS98, Flourochrome: BV 605, Catalouge #152208, Vendor: Biolegend). Anti-mouse CD101 (Clone: Moushi101, Flourochrome: PE, Catalouge #12-1011-82, Vendor: eBioscience). Anti-mouse CD117 (Clone: 2B8, Flourochrome: PE-Cy5, Catalouge #105810, Vendor: Biolegend). Anti-mouse CD19 (Clone: eBio1D3, Flourochrome: APC, Catalouge #17-0193-82, Vendor: eBioscience). Anti-mouse CD3 (Clone: 17A2, Flourochrome: APC, Catalouge #100236, Vendor: Biolegend). Anti-mouse CD49b (Clone: DX5, Flourochrome: APC, Catalouge #108910, Vendor: Biolegend). Anti-mouse CD16/32 (Clone: 93, Flourochrome: BV 711, Catalouge #101337, Vendor: Biolegend). Anti-mouse Ly-6C (Clone: AL-21, Flourochrome: PerCP Cy5.5, Catalouge #560525, Vendor: BD Bioscience). Anti-mouse FceRIa (Clone: MAR-1, Flourochrome: AlexaFlour 488, Catalouge #134329, Vendor: Biolegend). Anti-mouse Ly-6G (Clone: 1A8, Flourochrome: BUV 563, Catalouge #565707, Vendor: BD Bioscience). Anti-mouse I-A/I-E (MHC class II) (Clone: M5/114.15.2, Flourochrome: BUV 661, Catalouge #750280, Vendor: BD Bioscience). Anti-mouse TER119 (Clone: Ter-119, Flourochrome: BUV 661, Catalouge #750280, Vendor: BD

Bioscience). Anti-mouse Siglec-F (Clone: E50-2440, Flourochrome: PE-CF594, Catalouge #562757, Vendor: BD Bioscience). Anti-mouse IL-1β Pro-form (Clone: NJTEN3, Flourochrome: PE, Catalouge #12-7114-80, Vendor: eBioscience). Anti-human IL-1β (Clone: CRM56, Flourochrome: PE, Catalouge #12-7018-41, Vendor: eBioscience).

Antibodies used for western blot are: Caspase-1 (p20) (mouse; Adipogen #AG-20B-0042-C100), caspase-1 (p20) (human; Adipogen #AG-20B-0048-C100), cleaved-IL-1β (Asp117) (Cell Signaling Technology #52718), IL-1β (R&D Systems #AF-401), Ras (Abcam #52939), active Rac1 (NewEast Biosciences #26903). β-actin (Cell Signaling Technology #4970 or Santa Cruz Biotechnology #sc-47778) or Vinculin (Cell Signaling Technology #13901). Horseradish peroxidase (HRP)-linked anti-rabbit IgG or anti-mouse IgG were used as secondary antibodies (#7074 and #7076S Cell Signaling Technology).

These information are provided in the Supplementary methods.

Validation

To avoid antibodies off-target binding, minimise background and validate their specifity, antibodies were validated by titration. This was performed based on the recommended working concentration provided by the manufacturer. This validation was performed per experimental setup, conditions and cells type used.

Animals and other organisms

Policy information about studies involving animals; ARRIVE guidelines recommended for reporting animal research

Laboratory animals

C57BL/6 (H-2Kb, Thy-1.2) were purchased from Janvier Labs (France). Rosa26-Cre-ERT2 transgenic and LSL-KrasG12D knock-in mice were maintained on a C57BL/6N background and mated to generate Rosa26-Cre-ERT2; LSL-KrasG12D animals (local stock at the animal facility of University Medical Center Freiburg). Nlrp3-/- mice were obtained from the local stock at the animal facility of University Medical Center Freiburg. Rosa26-Cre-ERT2; LSL-KrasG12D were bred to Nlrp3-/- mice (both on C57BL/6 background) in the animal facility at the University of Freiburg, to generate the novel strain termed Rosa26-Cre-ERT2; LSL-KrasG12D; Nlrp3-/-. Rag2–/–γC–/– mice were obtained from the local stock at the animal facility of University Medical Center Freiburg. Mice were bred and housed under specific pathogen-free (SPF) conditions in the animal facilities of University Medical Center Freiburg (ZKF, Neurozentrum and IMMZ) and were used between 8 and 12 weeks of age at the time of the experiments. This information is provided in the Methods section.

Wild animals

The study did not involve wild animals.

Field-collected samples

The study did not involve samples collected from the field.

Ethics oversight

All mouse experiments were approved by the Federal Ministry for Nature, Environment and Consumers' Protection of the state of Baden-Württemberg, Germany (Protocol numbers: G-17/049, G-17/093, G-18/19, X-14/07H, X-15/09H, X-15/10A, X-18/10C).

Note that full information on the approval of the study protocol must also be provided in the manuscript.

Human research participants

Policy information about studies involving human research participants

Population characteristics

Human samples used in the study included patients with acute myeloid leukemia (AML), chronic myelomonocytic leukemia (CMML) or juvenile myelomonocytic leukemia (JMML), which either carry KRAS mutation, NRAS mutation or do not carry RAS mutations (non-mutant). JMML patients had a median age of 4 (range 0.5-7.6), 33.3% were females and 66.7% were males, all diagnosed with JMML and 100% carry a KRAS mutation.AML patients had a median age of 61 (range 25-80), 51.5% were females and 48.5% were males, all diagnosed with AML and 33.3% carry a KRAS mutation, while 66.7% are non-mutants.CMML patients had a median age of 75 (range 55-89), 39.1% were females and 60.9% were males, all diagnosed with CMML, 21.7% carry a KRAS mutation, 21.7% carry a NRAS mutation while 56.5% are non-mutants. This information is provided in the Supplementary Materials section.

Recruitment

Samples from JMML, CMML or AML patients of interest, who acquired written informed consent at University Medical Center Freiburg were only used.

Ethics oversight

Human sample collection and analysis were approved by the Institutional Ethics Review Board of the Medical center, University of Freiburg, Germany (protocol numbers 10024/13, 26/11, 509/16). Written informed consent was obtained from each patient. All analysis of human data was carried out in compliance with relevant ethical regulations

Note that full information on the approval of the study protocol must also be provided in the manuscript.

Flow Cytometry

Plots

Confirm that:

☒ The axis labels state the marker and fluorochrome used (e.g. CD4-FITC).

☒ The axis scales are clearly visible. Include numbers along axes only for bottom left plot of group (a 'group' is an analysis of identical markers).

☒ All plots are contour plots with outliers or pseudocolor plots.

☒ A numerical value for number of cells or percentage (with statistics) is provided.

Methodology

Sample preparation	Cells analysed by flow cytometry were either fresh mouse bone marrow (BM), spleen or blood cells; or mouse bone-marrow derived dendritic cells (BMDCs); or peripheral blood mononuclear cells (PBMCs) isolated from JMML, CMML or AML patients' blood samples. Based on experimental setup, cells were harvested according to the cell type, and counted using a haemocytometer. To examine cell viability and exclude dead cells, the LIVE/DEAD Fixable Dead Cell Stain kit (Molecular Probes) or the Zombie NIR Fixable Viability kit (Biolegend) were used. After 20 minutes incubation at 4°C, the cells were washed twice in FACS buffer and stained with the extracellular fluorochrome-conjugated antibodies in a final volume of either 50 or 100µL. After the staining, samples were washed twice to remove the unbound antibodies, and resuspended in a final volume of 200µL of FACS Buffer. For staining of intracellular antigens, the Cytofix/Cytoperm Fixation/Permeabilization (BD Biosciences) kit was used, according to the manufacturer's protocol. Briefly, cells were treated with Golgistop ™, a protein transport inhibitor containing monensin (1µl in 1.5 ml culture medium) and incubated at 37°C for 6 hours. Surface antigens were stained as described above. The cells were then washed, resuspended in Cytofix/Cytoperm solution and incubated for 20 min at 4°C. Subsequent washes were performed in 1x Perm/Wash buffer. Cells were stained with the intracellular antibody for 20 min at 4°C and washed, followed by two washes with Perm/Wash buffer and finally resuspended in a final volume of 200µL of FACS Buffer.
Instrument	The majority of data were acquired on a BD LSR Fortessa flow cytometer (BD Bioscience). Multiparameter high dimensional data were acquired on a FACSSymphony (BD Bioscience).
Software	The majority of data were analyzed using FlowJo (Flowjo 10.4 or 10.6, LLC) software. Multiparameter high dimensional data was compensated in FlowJo (V10.6), then live, single CD45+ cells were exported and analyzed using the R environment. Data was processed for FlowSOM clustering as described. For dimensionality reduction the UMAP package was used.
Cell population abundance	The relevant cell population was sorted using BD FACSARIA III cell sorter. The gating strategy was determined using unstained controls, single stains and fluorescence minus one (FMO) controls. The sorted cells were further analyzed using flow cytometry for determination of purity. All the post-sort fractions were at least 90% pure. This information is provided in the Suplelementary Figures.
Gating strategy	For all experiments, proper controls and compensation controls were considered. Cells were first gated using FSC-A/SSC-A, following FSC-A/FSC-H and SSC-A/SSC-H to exclude doublet cells. After, dead cells were excluded using gating on Amcyan (LIVE/DEAD™ Fixable Aqua Dead Cell Stain Kit, for 405 nm excitation). Subsequently, the desired parameters were gated. Unstained controls, single stains and fluorescence minus one (FMO) controls were used for appropriate gating on desired parameters and differentiation between positive and negative cells. The gating strategy of all relevant experiments is provided in the supplementary figures.

☒ Tick this box to confirm that a figure exemplifying the gating strategy is provided in the Supplementary Information.
